# Monitoring multiple parameters in complex water scenarios using a low-cost open-source data acquisition platform

**DOI:** 10.1016/j.ohx.2023.e00492

**Published:** 2023-11-28

**Authors:** Steven Martinez Vargas, Alejandro J. Vitale, Sibila A. Genchi, Simón F. Nogueira, Andrés H. Arias, Gerardo M.E. Perillo, Agustín Siben, Claudio A. Delrieux

**Affiliations:** a Instituto Argentino de Oceanografía (IADO), CONICET-Universidad Nacional del Sur (UNS), B8000FWB, Bahía Blanca, Argentina; b Departamento de Ingeniería Eléctrica y de Computadoras, UNS, Bahía Blanca, Argentina; c Departamento de Geografía y Turismo, UNS, Bahía Blanca, Argentina; d Departamento de Ingeniería, UNS, Bahía Blanca, Argentina; e Departamento de Química, UNS, Bahía Blanca, Argentina; f Departamento de Geología, UNS, Bahía Blanca, Argentina; g Instituto de Ciencias e Ingeniería de la Computación, CONICET-UNS, Bahía Blanca, Argentina

**Keywords:** Data Acquisition Platform, EMAC-USV, Water Quality Parameters, Bathymetry, Low-Cost, Open-Source

## Abstract

Water monitoring faces challenges that are driven by the infrastructure, protection, financial resources, science and innovation policies, among others. A modular, low-cost, fully open-source and small-sized Unmanned Surface Vessel (USV) called EMAC-USV (EMAC: Estación de Monitoreo Ambiental Costero), is proposed for monitoring bathymetry and water quality parameters (i.e. temperature, suspended solids concentration and hydrocarbon concentration) in complex water scenarios. A detailed description of each part of the platform as well as all electronic connections and functioning is presented.The field works were carried out in two small waste stabilization ponds and in a portion of the main tidal channel of the Bahía Blanca port. The EMAC-USV is the result of a cautious design, regarding the balancing performance, communications, payload capacity, among others.

## Graphical abstract

**Figure:**
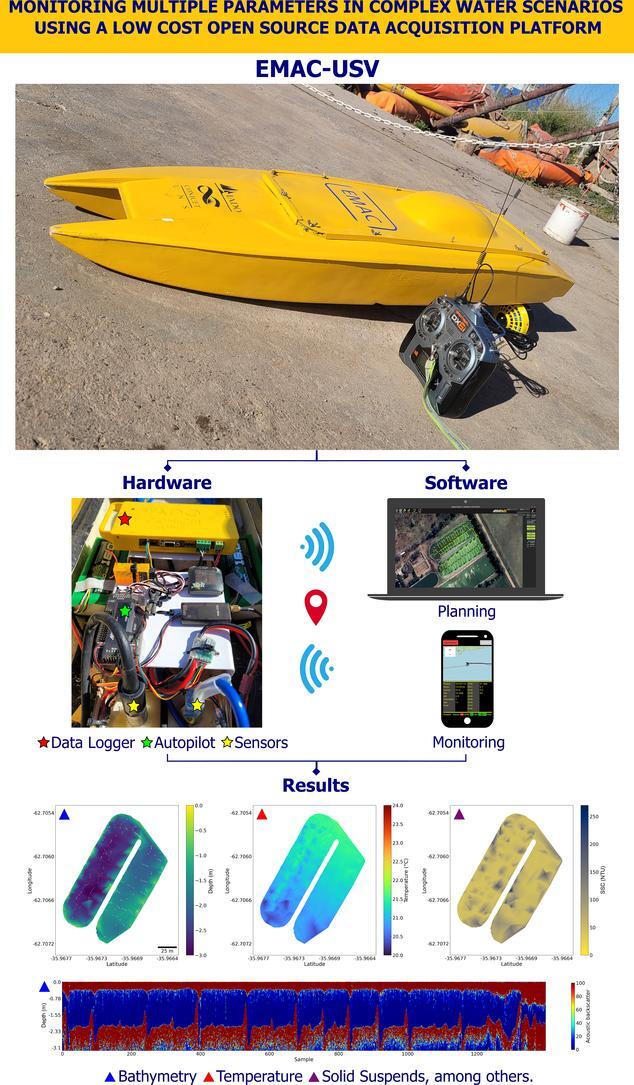

**Table:** 

Hardware name	EMAC-USV
Subject area	Engineering and materials science; Environmental, planetary and agricultural sciences
Hardware type	Field measurements and sensors
Closest commercial analog	CEE-USV
Open source license	GNU GPL v3
Cost of hardware	USD 2247
Source file repository	Mendeley Data

## Hardware in context

Water is a primary necessity for all living organisms. The focus point of water management can be understood as a balance between different demands for water, from sanitation to energy generation to ecosystem protection [1]. It is a prerequisite to carry out an adequate monitoring of water parameters in order to gain knowledge and make appropriate decisions and actions [2]. However, water monitoring is a task that usually faced with many challenges. These challenges include: intrinsic characteristics of the study field to be monitored (for example, hydrodynamics, area and depth) [3], [4], financial resources [5], [6], science and innovation policies [7], [8], among others.

The technological development in unmanned platforms is an emerging field of considerable interest for researchers in water resources. Yet at the beginning of the 2010 s, de Sousa and Gonçalves [9] highlighted that future generations of unmanned vehicles should reflect trends in increased levels of autonomy, lower cost, longer endurance and networking capabilities. Several issues such as communication [10], [11], engineering [12], [13], navigation control [14], [15], composite materials [16] and power systems [17], [18], [19] are being widely investigated for purposes of water monitoring.

Recently, interesting studies discussed about housing developments. For instance, Jo et al. [20] presented a low-cost, small and open-source platform for measuring near-surface water quality. Carlson et al. [15] developed an affordable and portable unmanned surface vehicle that can obtain bathymetry and ocean current measurements in dangerous environments such as shallow and rocky areas under safe conditions. Madeo et al. [21] realized a surface vehicle exploiting low-cost off-the-shelf components that was crucial to be replicated in large quantities in order to set up a sort of “Social Sensor Network”.

The commercial unmanned surface platforms are expensive (from several thousand to several million USD depending on its on-board sensors) to be used for research purposes [22] mainly in developing countries. In addition, many existing platforms are no fully open-source, which does not allow customization of end-users’ requirements [20]. In this article a modular, low-cost, fully open-source and small-sized Unmanned Surface Vessel (USV) called EMAC-USV (EMAC: Estación de Monitoreo Ambiental Costero) is proposed. A complete description of each part of the platform as well as all electronic connections and functioning is presented. On the other hand, an important contribution is to provide detail on the design and development of a low-cost and flexible data logger with its open-source custom firmware, that can be easily replicated. For this work, bathymetry and water quality parameters (i.e. temperature, suspended solids concentration (SSC) and hydrocarbon concentration (HC)) were monitored in complex water scenarios: 1) Small (and shallow) waste stabilization ponds (Buenos Aires province, Argentina) and 2) A portion of the main tidal channel of the Bahía Blanca port (Bahía Blanca estuary, Argentina). The EMAC-USV is the result of a cautious design, regarding the balancing performance, communications, payload capacity among others.

## Hardware description

**Fig. 1:**
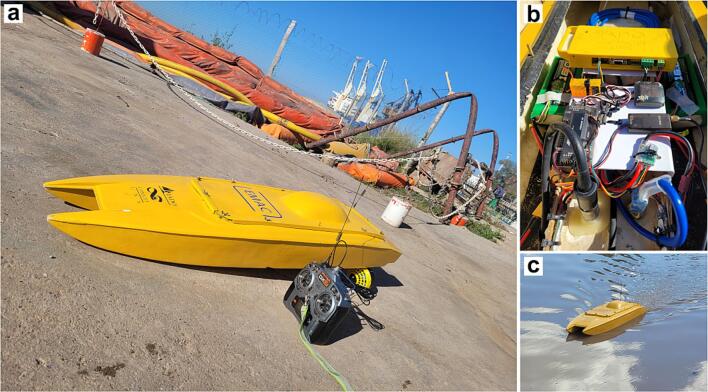
Different views of the EMAC-USV: (a) The USV prior to putting it into operation, (b) inner parts of the USV, and (c) USV operating.

Most electronic and mechanical parts of the EMAC-USV were designed and developed by a team of researchers from the Instituto Argentino de Oceanografía (IADO-CONICET, Bahia Blanca, Argentina) (Fig. 1). The platform, which will be described below in detail, was greatly improved in the last few years based mainly on physical and kinematic constraints inherent to complex body waters. Early bathymetry surveys were conducted in shallow lakes [23], [24] and in a tidal channel [25], [26].

### Vessel architecture and design constraints

**Fig. 2:**
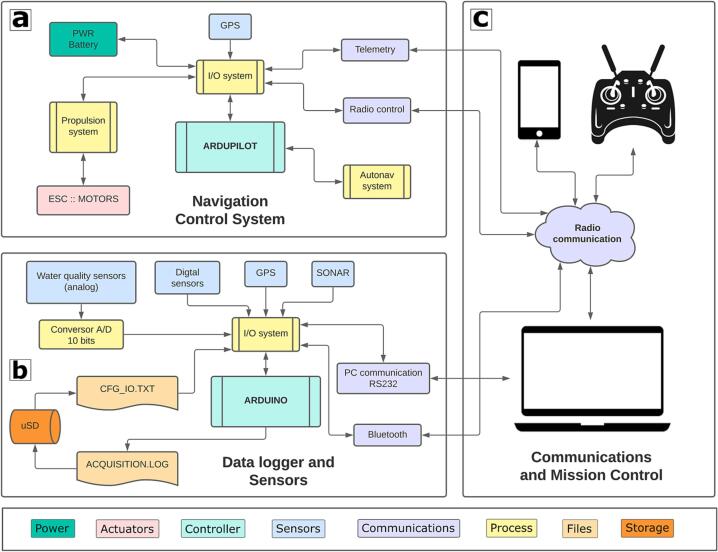
Flow diagram describing the software/hardware architecture of the EMAC-USV. The diagram can be divided into three principal parts: (a) navigation, (b) data logger and sensors, and (c) communication.

**Fig. 3:**
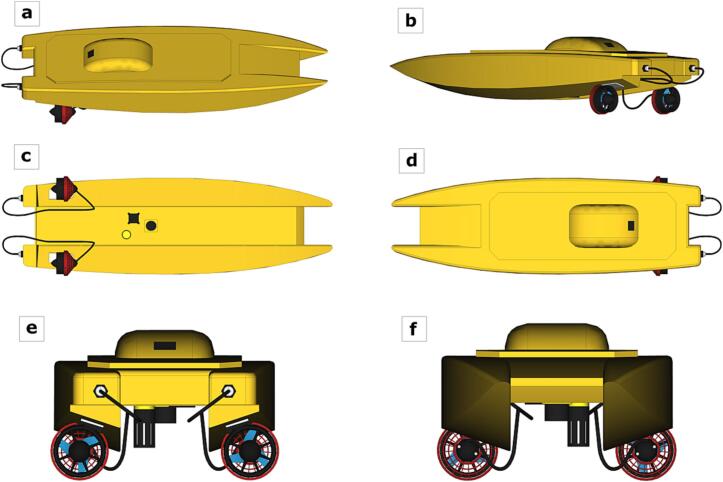
Rendered design of the EMAC-USV at different views: (a,b) lateral views, (c) bottom view, (d) top view, (e) back view, and (f) front view.

A flow diagram describing the software/hardware architecture of the proposed platform is shown in Fig. 2. Technical specifications and rendered design of the EMAC-USV are shown in Table 1 and Fig. 3, respectively. Structurally, the vessel is a catamaran hull of 1.30 x 0.35 x 0.30 m (Fig. 3). The design of the platform is based on physical and kinematic constraints. For instance, a low draft (approx. 0.12 m, Fig. 3b) results helpful to achieve better flatness. The hull is built of fiberglass and is covered by epoxy resin, which offers improved resistance to fatigue and durability [27]. The hull contains all the electronics modules, inner sensors, power package and motor. The weight of the platform including a standard battery bank is 8.6 kg; it has a high payload capacity of 5 kg (Table 1). The power supply system consists of a bank of two 4S Lithium Polymer (Li-Po) batteries of 16,000 mAh (Table 1). In the actual version of the EMAC-USV, two horizontal fixed thrusters (T200 model) were mounted on the rear portion of the hull in order to differentially control speed and direction (Table1, Fig. 3e,f). These commercial thrusters are low-cost and energy efficient.

**Table 1:** Technical specifications of the EMAC-USV.

USV Specification	Description
**Technical Specifications**	
Dimension	1 x 0.45 x 0.27 m
Vehicle weight	8.6 kg (Standard battery bank)
Payload weight	5 kg
Storage capacity	6.2 L
Cruising speed	1.5 m s^−1^
Standard battery bank	4S − 16,000 mAh x 2
Extended battery bank	4S − 16,000 mAh x 4
Standard operation time	6 h (Standard battery bank: 16,000 mAh x 2)
Auto pilot	ArduPilot 2.5
Thruster	T200-THRUSTER-R2-RP (@ 16 V/24A − 390 W) x 2
GPS	3DR uBlox (5 Hz update rate - with HMC5883L digital compass module)
Radio telemetry modem	RDF900, 900Mhz, 1 W, max. distance: 2 to 5 km depending on the base station height
Data logger	8 analog inputs and 3 digital inputs, SD card up to 64 Gb, Bluetooth connection
Sonar	Ping Sonar
**Water quality sensors**	
Temperature	Accuracy: ±0.1 °C; validated measurement range: −5–35 °C
Solid Suspends	Accuracy: ±3 %; validated measurement range: 0–200 NTU
**Working conditions**	
Air temperature range	−10 to 50 °C
Wind speed tolerance	Up to 12 m s^−1^ (calm waters)
Wave height tolerance	Up to 0.5 m
Water flow tolerance (opposite flow direction)	Up to 0.5 m s^−1^
Minimum turning radius	2.5 m

The autonomy of the platform according to the battery capacity is approximately 6 h. The platform should be operated under a set of optimal working conditions which were estimated based on several field tests (Table 1 lower part). Among the most important conditions are: wind speed less than 12 m s^−1^ and wave height less than 0.5 m.

### Data logger and sensors

The EMAC-USV is equipped with sensing units which are mostly deployed in some Argentine lakes and the sea [28], [29].

#### Data logger

**Fig. 4:**
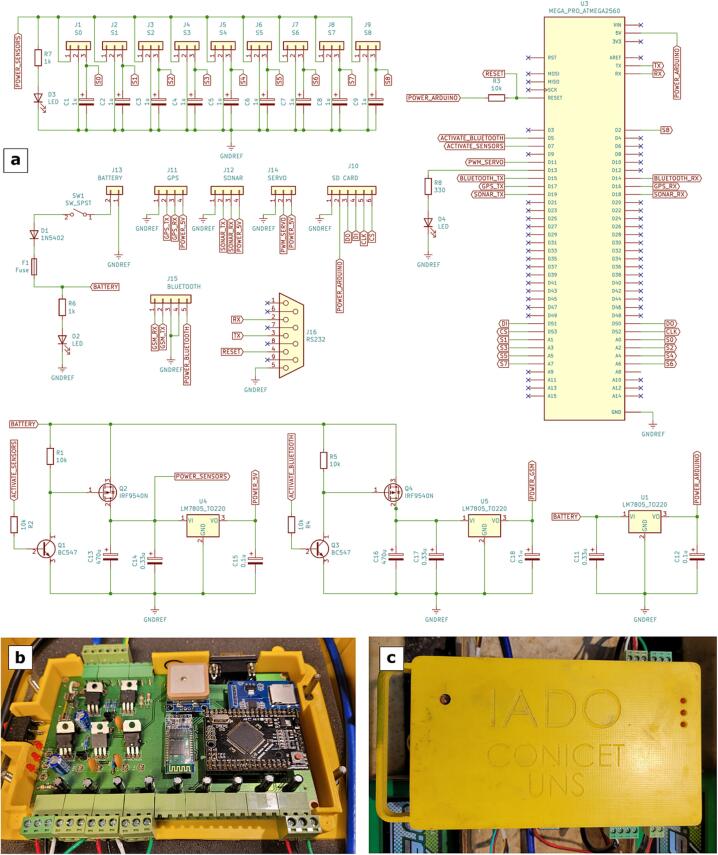
Data logger: (a) the schematic circuit, (b) the circuit board implementation, and (c) data logger box.

**Fig. 5:**
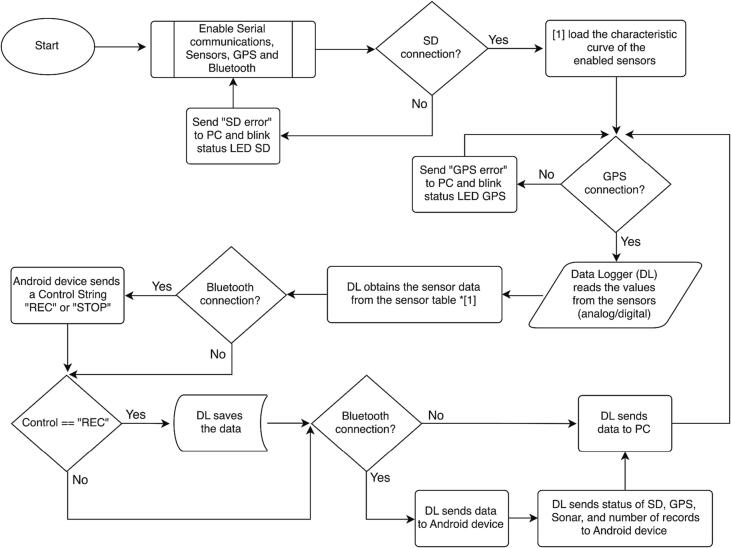
Flow diagram describing the operation of the data logger.

Table 2 summarizes the technical description of the designed data logger. The data logger is a compact case (0.20 x 0.11 x 0.03 m) that was 3D printed (1b). The device consists of a dual layer single board including GPS, communication modules (Bluetooth and GPRS modem -optional-), data storage and power management (Table 2, Fig. 4a,b). The data logger has also a RS232 communication port to interface to a PC for data debugging and firmware update. The data logger has 3 digital inputs and 8 analogical inputs (10-bit resolution) allowing the integration of commercial sensors with analogical or serial outputs. It is based on Arduino ATmega2560 microcontroller board. The circuit board implementation and the schematic circuit can be seen in Fig. 4b and c, respectively. The data is stored on a micro-SD card that supports various memory sizes (from 4 up to 64 Gb). A flow diagram describing the operation of the data logger is shown in Fig. 5.

**Table 2:** Description of the main technical characteristics of the data logger.

Parameters	Description
Operating temperature range	−40 to 70 °C
Analog inputs	8 single ended (expandable to 16)
Pulse counters digital inputs	1 (expandable to 2)
Communications ports	RS-232
Max. sample rate	10 Hz (without echosounder), 3 Hz (with echosounder)
GPS	U-blox NEO-6
Input limits	+5 V
ADC	10 bits
Power range	9 – 24 V
Remote communication technology	Bluetooth/GPRS/RADIO
Communication range	Bluetooth: 50 m GPRS: 15 km (Dependent of the service provider) RADIO: 2 to 5 km (Dependent of the site)
Max. current supply	3 A
Max. storage	64 Gb

#### Single beam echosounder

The EMAC-USV is equipped with a compact ultrasonic Ping sonar manufactured by Blue Robotics at a competitive price. Recent studies successfully used this device [30], [31], [32]. This single beam sonar works at a range of 0.3–100 m and a beam width of 25 degrees, with a frequency of 115 kHz. The accuracy of the sensor is 0.5 %. Besides the depth, the sonar provides the full echo response in order to carry out analysis of water column as well as bottom sediment. The latter was programmed to be recorded by the data logger.

#### Temperature and suspended solids concentration

The temperature and SSC sensors, which were designed by the second author, are integrated into a single sensor element. The temperature and SSC sensors employ NTCLE203E3103SB0 thermistor and optical backscatter sensor, respectively [28]. The sensor body is built in stainless steel and the head is a 3D printed piece. SST sensor uses a Microchip PIC12F675 microcontroller to generate the 10 kHz infrared pulse OPB730F (reflective object sensor). For the analog output, the backscatter signal is amplified and processed by means of a precision rectifier circuit, using integrated circuit technology (TL082) [28]. The accuracy is ± 0.1 °C (temperature) and ± 3 % (SSC); the response time of the sensor is lower than 1 s.

#### Hydrocarbon concentration

The platform is also equipped with a HC sensor (C-FLUOR model) manufactured by Turner Designs. It consists of a single wavelength in situ fluorescence with an analog output signal of 0 up to 5 V. This sensor uses a blue excitation working from 0 to 200 ppb. The response time of the sensor is lower than 1 s.

### Control system and communication

**Fig. 6:**
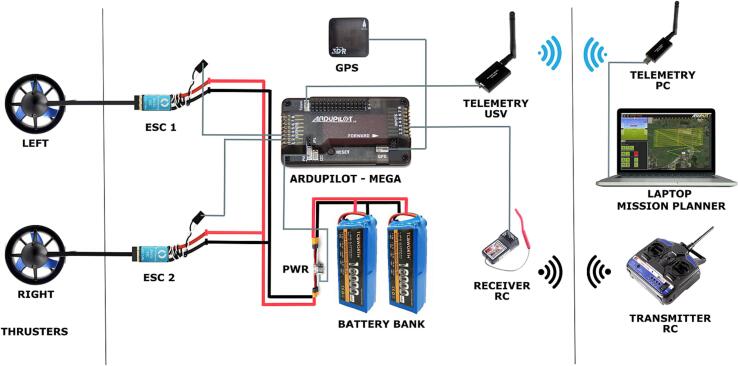
Architecture of the control system and communication of the EMAC-USV.

An Ardupilot Mega board (APM) is used as the IMU autopilot system allowing for autonomous navigation (Fig. 6). The wireless communication between the operator and the USV is based on two modes: RDF900 + at 900 MHz bidirectional telemetry and radio control at 2.4 GHz frequency with a minimum of 4 channels (Fig. 6). The platform follows a mission from preprogrammed waypoints using bidirectional telemetry with wireless modules. The EMAC-USV uses the 3DR ublox LEA-6 GPS module that provides enough performance, accuracy and compass. The data logger is linked to the Mission Planner software to monitor the echosounder profile during the field work.

## Design files summary

Table 3

**Table 3:** Hardware files.

Design file name	File type	Open source license	Location of the file
**Hull**			
USV_3D.STEP -Fig. 3 -	CAD	GPLv3	Mendeley_Data
**EMAC-USV architecture**			
Ardupilot-system.png -Fig. 6 -	PNG	GPLv3	Mendeley Data
EMAC-architecture.png -Fig. 2 -	PNG	GPLv3	Mendeley Data
**Data logger**			
EMAC-USV-Datalogger.pdf -Fig. 4 -	PDF	GPLv3	Mendeley Data
Datalogger.pro	Kicad Project	GPLv3	Mendeley Data
Datalogger.sch	Kicad schematic	GPLv3	Mendeley Data
Datalogger.kicad_pcb	Kicad pcb layout	GPLv3	Mendeley Data
**Sensors**			
Temperature			
Temperature.pdf	PDF	GPLv3	Mendeley Data
Temperature.kicad_pro	Kicad Project	GPLv3	Mendeley Data
Temperature.kicad_sch	Kicad schematic	GPLv3	Mendeley Data
Temperature.kicad_pcb	Kicad pcb layout	GPLv3	Mendeley Data
Suspended solids concentration			
SSC.pdf	PDF	GPLv3	Mendeley Data
SSC.kicad_pro	Kicad Project	GPLv3	Mendeley Data
SSC.kicad_sch	Kicad schematic	GPLv3	Mendeley Data
SSC.kicad_pcb	Kicad pcb layout	GPLv3	Mendeley Data

Table 4

**Table 4:** Software files.

Design file name	File type	Open source license	Location of the file
**Data logger**			
Datalogger-Diagram.png -Fig. 5-	PNG	GPLv3	Mendeley Data
Sketches			
EMAC-Datalogger.ino	Arduino sketch	GPLv3	Mendeley Data
data_file_sensors.h	Arduino sketch	GPLv3	Mendeley Data
gps.h	Arduino sketch	GPLv3	Mendeley Data
sonar.h	Arduino sketch	GPLv3	Mendeley Data
starter_main.h	Arduino sketch	GPLv3	Mendeley Data
main.h	Arduino sketch	GPLv3	Mendeley Data
bluetooth.h	Arduino sketch	GPLv3	Mendeley Data
print_data.h	Arduino sketch	GPLv3	Mendeley Data
SD-files			
EMACConf.ini -Fig. 7i -	Txt (sensors configuration)	GPLv3	Mendeley Data
obs001.sen	Txt (sensors/SSC)	GPLv3	Mendeley Data
tem007.sen	Txt (sensors/Temperature)	GPLv3	Mendeley Data
cfl000.sen	Txt (sensors/HC)	GPLv3	Mendeley Data
**Mobile app**			
Dashboard.png -Fig. 8 -	PNG	GPLv3	Mendeley Data
Dashboard.apk	APK	GPLv3	Mendeley Data
Dashborad.aia	App Inventor sketch	GPLv3	Mendeley Data

## Bill of materials summary

Table 5

**Table 5:** Materials summary.

Designator	Component	Number	Cost per unit -currency (USD)	Total cost -currency(USD)	Source of materials	Material type
**USV**						
Hull -Fig. 1a,3 -	EMAC hull	1	350	350	Local manufacturing	Fiberglass
Thruster -Fig. 7b -	T200	2	200	400	https://bluerobotics.com/store/thrusters/t100-t200-thrusters/t200-thruster-r2-rp/	Other
ESC -Fig. 7a -	Basic ESC	2	36	72	https://bluerobotics.com/store/thrusters/speed-controllers/besc30-r3/	Other
Battery -Fig. 7a -	Lipo Battery 16000mAh 4S 12C 14.8 V Liperior	2	170	340	https://rcbattery.com/liperior-16000mah-4s-12c-14-8v-lipo-battery-with-xt90-plug.html	Lithium polymer
Radio Control(*) -Fig. 1a -	Flysky FS-i6X	1	68	68	eBay_link	Other
ArduPilot with GPS(*) -Fig. 6 -	Pixhawk PX4	1	149	149	https://rb.gy/0p5ta	Other
Radiotelemetry Kit(*) -Fig. 6-	–	1	128	128	https://rb.gy/ask94	Other
**Data Logger and sensors**						
Data Logger -Fig. 4-	EMAC data logger	1	200	200	Local manufacturing	Other
Sonar -Fig. 7b-	Ping Sonar	1	390	390	https://bluerobotics.com/store/sonars/echosounders/ping-sonar-r2-rp/	Other
T&SSC Sensor -Fig. 7a,b-	EMAC	1	150	150	https://www.degruyter.com/document/doi/10.1515/pac-2018-0508/html?lang=enelectronics.com/TTElectronics/media/ProductFiles/Datasheet/OPB710-730.pdf	Stainless steel body
HC sensor -Fig. 7a,b-	C-FLUOR	1	3200	3200	https://www.turnerdesigns.com/c-fluor-submersible-probes	Other

(*) Discontinued parts; they were replaced by similar others available in the current table.

## Build instructions

Descriptions of our contributions will be detailed below. For the built instructions belonging to commercial parts of the proposed platform, it must follow their own specifications and capabilities.

**Hull built.** The hull was built by local builders based on standard materials and measurements (i.e. aspect ratio) for the optimal navigation conditions (Fig. 1a;3, Table 1). Additionally, after several positive tests, a 3D rendered model of the hull from the CAD tool was created (File name: USV_3D.STEP) which serves as a digital twin. Therefore, the hull could be replicated by using fiberglass or plastic materials such as acrylonitrile butadiene styrene (ABS) used in the 3D printer, and polyvinyl chloride (PVC) used in the rotomolding.

**Fig. 7:**
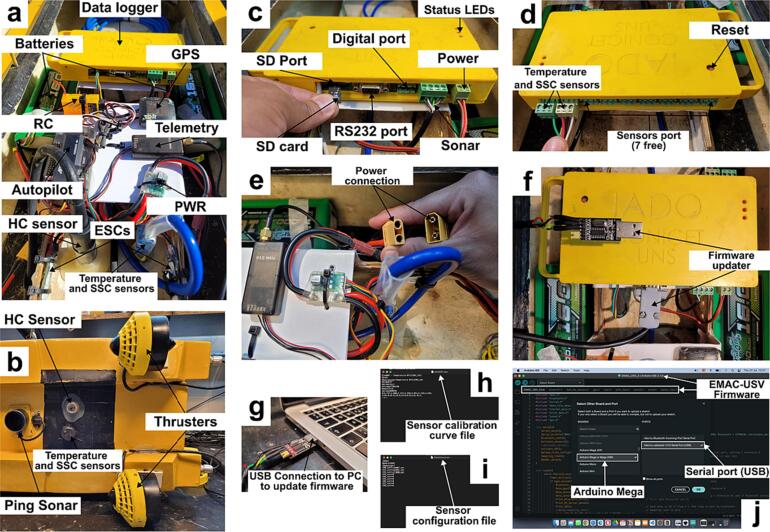
Assembly view of all EMAC-USV components and firmware update. (a) main internal components, (b) thrusters and sensors, (c) data logger configuration ports, (d) data logger sensor port, (e) power connection, (f) data logger firmware update mode, (g) firmware update mode through pc connection, (h - i) sensor files, and (j) firmware updater via Arduino IDE.

**Data logger and sensors.** The data logger and some water quality sensors (i.e. temperature and SSC) previously described in the hardware description section were self developed by the authors based on an open and low-cost concept (Fig. 7a,b). Firstly, for data logger and sensors, printed circuit boards (PCB) interfacing electronic modules were designed using KiCAD software. The electronic components were soldered to printed PCBs, and then the final boards were tested. Each board contains a microcontroller as the main processing unit which was properly programmed. Once the components of the data logger are assembled, the firmware is uploaded through the serial port (Fig. 7c-g). The firmware files (EMAC-Datalogger.ino) are compiled and uploaded to the datalogger using the Arduino IDE (Fig. 7j). The sensors were fully filled with epoxy resin to make it highly waterproof [28]. Additionally, the sensors were calibrated in the laboratory in order to find a correlation between the real values and sensor output. The response curve of the temperature sensor is provided by the manufacturer's data sheet which contains electrical information of resistance of the thermistor at different temperatures. This response curve was validated at 5, 15 and 25 °C using a high-accuracy thermometer (Valeport miniCT, accuracy: ±0.01 °C) immersed in the water solution. In the case of the optical baskatter sensor, the calibration was carried out as follows: First, a known value of pure water was considered as zero for the sensor; then, formazin standard solutions at 20 and 200 NTU were used; finally, a response curve of voltage vs. NTU from 0 to 200 NTU was obtained. In order to guarantee a complete submersion of the sensor at least 1 L solution was used. In addition, a black recipient was used to avoid any reflection. All the PCBs, circuits and calibration curves are available as supplementary material (extension file: *.kicad_pro, *.kicad_sch, *.kicad_pcb).

**Mounting.** Because of the high volumetric capacity of the hull (6.2 L), there is flexibility in the distribution of the inner parts of the platform. For placing the parts, it is key to control both the center of mass and the center of buoyancy, which must occur at the same relative location. In this proposed platform, the battery bank (one on each side) and the remaining parts were located at the central part (Fig. 1b; 7a). The datalogger and the different components of control and communications system (autopilot, GPS, RC, telemetry, PWR and ESCs) were positioned on a support raised from the floor of the hull to avoid water leaks (Fig. 7a). On the other hand, with the aim of achieving a minimum optimal navigation condition and a low-cost of implementation, two thrusters were employed which were precisely positioned (Fig. 3c,e; 7b).

## Operation instructions

**Step 1: Navigation path planning.** Firstly, the user defines the path in terms of a set of waypoints by using the Mission Planner software. The path planning is then uploaded to the Ardupilot using the above mentioned software. The website [33] includes an overview of the Mission Planner implementation (installation, Ardupilot firmware, connections, mission planning, etc.).

**Step 2: Preparation, transportation and navigation.** The Li-Po batteries must be 100 % fully charged. The platform must be transported to the field horizontally to prevent the parts from hitting each other as well as from disconnecting. Once in the field, the user inspects the inner parts and connections and then closes the lid and the bolts to avoid water leaks. Before starting the navigation, a general inspection of the monitoring zone should be carried out because emergent obstacles can occlude the path; if the latter is true, corrections of the planning must be made. Regarding the care of the user, precautions against possible risks caused by thrusters must be taken into account.

**Fig. 8:**
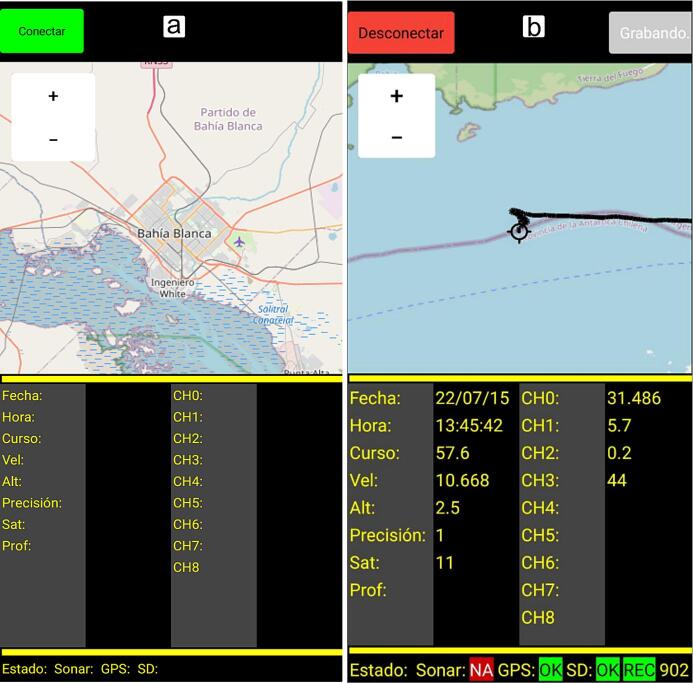
View of the EMAC Monitor data logger software. (a) stand by mode and (b) recording data.

**Step 3: Real-time data monitoring and data record.** The EMAC Monitor data logger software, developed by the authors, is a user-friendly Android application via Bluetooth that allows to visualize the sensors and the path planning, check the GPS status, enable/disable the data logging, and to set a wide range of configurations (Fig. 8). Each sensor is configured by editing the associated text file that contains its calibration curve which is stored on the micro-SD card in the sensor directory (Fig. 7c,h,i). Finally, the recorded data is saved into a file; each file contains the GPS location data, time, date, and the sensed data itself.

**Step 4. Cleaning of the platform.** Cleaning of the external parts of the platform such as hull, thrusters and water quality sensors is required after its use through the employment of distilled water.

## Validation and characterization

In this study, two complex water scenarios (two small waste stabilization ponds and a portion of a tidal channel) were considered to have a complete overview of the performances of the proposed USV.

### Scenery #1: Two small waste stabilization ponds

**Fig. 9:**
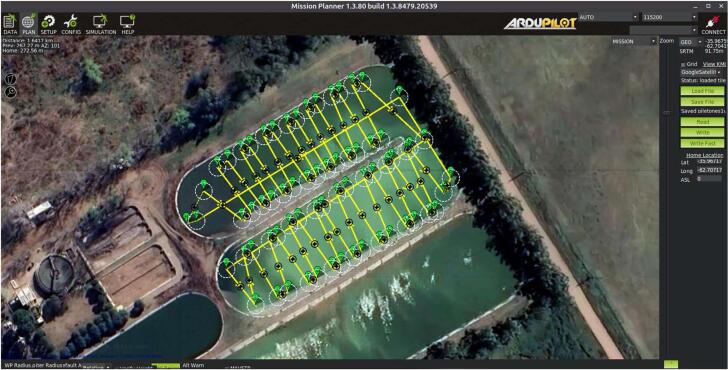
Example of the designed trajectory in scenery #1 (pond 1) using the Mission Planner software.

**Fig. 10:**
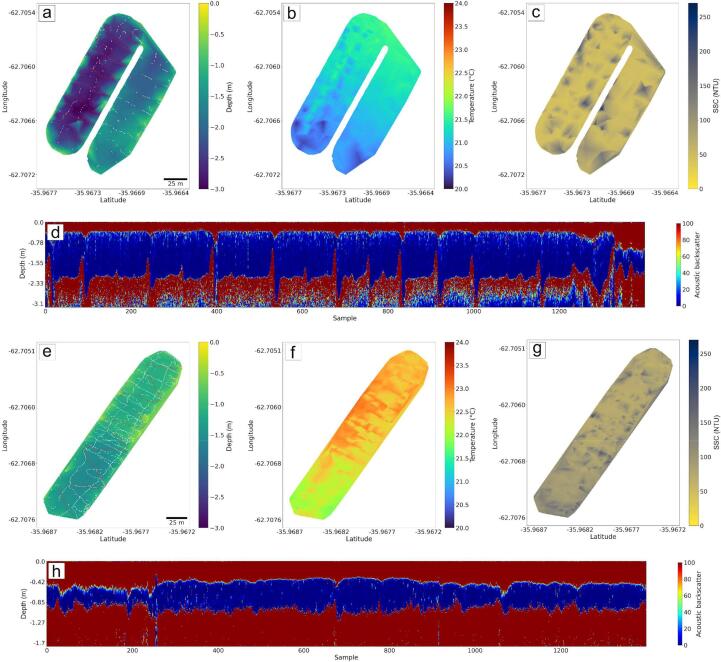
Pond 1 (left and right parts) (a-d) and pond 2 (e-h). (a,e) USV trajectory and bathymetric maps, (b,f) temperature maps, (c,g) suspended solids concentration (SSC) maps, and (d,h) full echo response data belonging to a transect of each pond (see red line in Figs. a and e). (For interpretation of the references to colour in this figure legend, the reader is referred to the web version of this article.)

A set of measurements of quality parameters and bathymetry were carried out in two small waste stabilization ponds located in the west of the Buenos Aires province (Argentina), in October 2022. The description of the ponds, USV navigation and weather conditions is presented in Table 6. In this campaign, the USV was operated automatically, but occasionally it was controlled manually to avoid the obstacles into the ponds such as grass or floating garbage (Fig. 9). In this study the well known linear interpolation method for mapping the water parameters and bathymetry was employed. The USV trajectory, the bathymetric, temperature and SSC maps as well as the full echo response data belonging to a transect of each pond are shown in Fig. 10. The EMAC-USV produced clear patterns of distribution. As can be expected, the depths increase towards the center reaching −3 and −1.7 m in ponds 1 -left part- and 2, respectively (Fig. 10a,e). The ponds showed a similar pattern of temperature distribution in response to the liquid effluents entering into the ponds, which have their maximum peak shortly before the measurement (Fig. 10b,f). In particular, a marked difference of 1.5–––2 °C in water temperature was observed in pond 1 (Fig. 10b). The details of the full echo response are influenced by the bottom characteristics of the ponds which are covered by a waterproofing layer based on gravel. This latter gives abrupt changes between the two interfaces (Fig. 10d,h).

**Table 6:** Description of the monitored ponds, USV navigation and weather conditions.

	Pond		
	1 -right part-	1 -left part-	2
Area (m^2^)	6322	6560	14,722
Major axis length (m)	153	156	274
Minor axis length (m)	45	44	55
Mean depth (m)	2.1	1.5	1.1
Total travel distance (m)	1236.8	1814.6	3667.9
Mean velocity of navigation (m/s)	0.76	0.98	0.85
Surveying time (min)	27.0	30.6	71.9
Weather conditions	-Wind: Mean vel. = 10 m s^−1^, Mean dir. = 210° -Mean air temp. = 25 °C

### Scenery #2: Canal Principal (Bahia Blanca estuary)

On-board measurements of bathymetry as well as water quality parameters such as temperature, SSC and HC were carried out in the inner part of the Canal Principal of the Bahia Blanca estuary (Argentina), in May 2023. This is a navigation channel with a depth of approximately 13 m. Due to the traffic conditions at the site of measurements and to the legal regulations, the USV navigation was manually controlled.

**Fig. 11:**
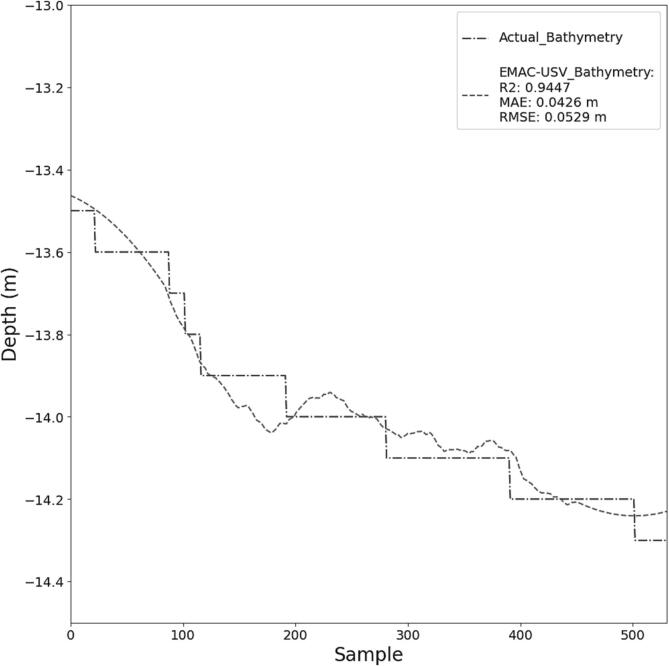
Assessment of our bathymetric data using high quality wideband multibeam echosounder data (Sonyc2020 model).

Our bathymetric data were assessed against high-quality wideband multibeam echosounder data (Sonyc2020 model) provided by the CGPBB (Fig. 11). Corrections for tides at measurement time were made as part of postprocessing. In order to statistically analyze the data (i.e. depth), accuracy measures such as root mean square error (RMSE), mean average error (MAE) and coefficient of determination (R^2^) were used. As can be seen from Fig. 11, the error is nearly independent from the depth range, reaching values from 0.04 (MAE) to 0.05 m (RMSE). The results show a clear positive correlation (R^2^ = 0.945) between our measured data and the reference data.

**Fig. 12:**
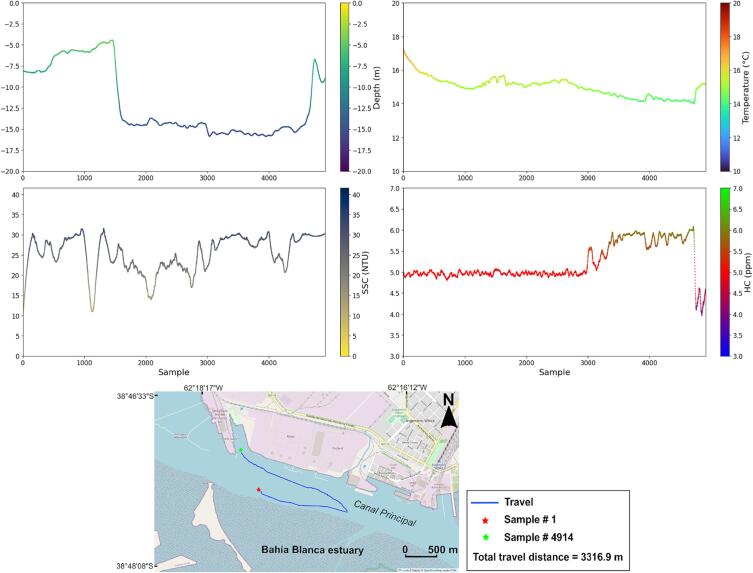
Measurements of bathymetry, temperature, suspended solid concentration (SSC) and hydrocarbon concentration HC in the inner part of the Principal Channel of the Bahia Blanca estuary (Argentina).

The EMAC-USV was capable of performing high resolution measurements (Fig. 12). The total sample number was 4914, covering a distance of 3317 m. According to the plots, most of water quality parameters, at difference of HC, seem to respond to depth variations.

## Conclusion

Water monitoring faces challenges that are different from those faced in air or ground environments; these include: infrastructure, protection, financial resources, science and innovation policies among others. This study describes an unmanned surface platform based on an open-source concept and low-cost. The total cost of the proposed USV platform is about USD 2300 without considering the commercial HC sensor. The cost is much lower than commercially available platforms and, therefore, it is suitable for the water science community, even more for developing countries.

An important advantage of the proposed platform is its ability to cover very shallow water bodies. The deepest part of the platform hull only protrudes less than 0.12 m below the water surface. Another advantage of the EMAC-USV is that it is modular, where most parts of the USV can be replaced in the field adding robustness to the platform operation. In addition, the inner parts of the platform can be easily scalable and transferred to a larger offshore platform or vessel for monitoring highly dynamic environments.

The major limitation of the EMAC-USV may be its cruising speed (1.5 m s^−1^) when it is compared with commercial expensive vehicles, which can for example reach 2.5 [34] and 4 m s^−1^ [35]. It should be mentioned that the latter maximum values could facilitate travel between distant sampling points. As a future improvement of the proposed platform, a RGB video camera will be added to achieve real-time captioning of the surrounding operation zone and, therefore, to avoid obstacles, especially in complex sceneries. In addition, a modular automatic water sampler should be well integrated due to the extra volume storage of the hull, which is approximately 40 %.

## CRediT authorship contribution statement

**Steven Martinez Vargas:** Conceptualization, Methodology, Hardware, Software, Validation, Visualization, Formal analysis, Data curation, Writing – review & editing, **Alejandro J. Vitale:** Conceptualization, Methodology, Hardware, Software, Validation, Formal analysis, Investigation, Resources, Supervision, Funding acquisition. **Sibila A. Genchi:** Conceptualization, Validation, Data curation, Writing – original draft, Writing – review & editing, Visualization. **Simon F. Nogueira:** Mechanical Design, 3D printing. **Andrés H. Arias:** Investigation. **Gerardo M.E. Perillo:** Investigation. **Agustín Siben:** Software. **Claudio A. Delrieux:** Investigation.

## Declaration of competing interest

The authors declare that they have no known competing financial interests or personal relationships that could have appeared to influence the work reported in this paper.
