# Supplementary figures and images for: Monitoring multiple parameters in complex water scenarios using a low-cost open-source data acquisition platform
Source: HardwareX. 2023 Nov 28;16:e00492. doi: 10.1016/j.ohx.2023.e00492 (PMC10749909; doi:10.1016/j.ohx.2023.e00492)

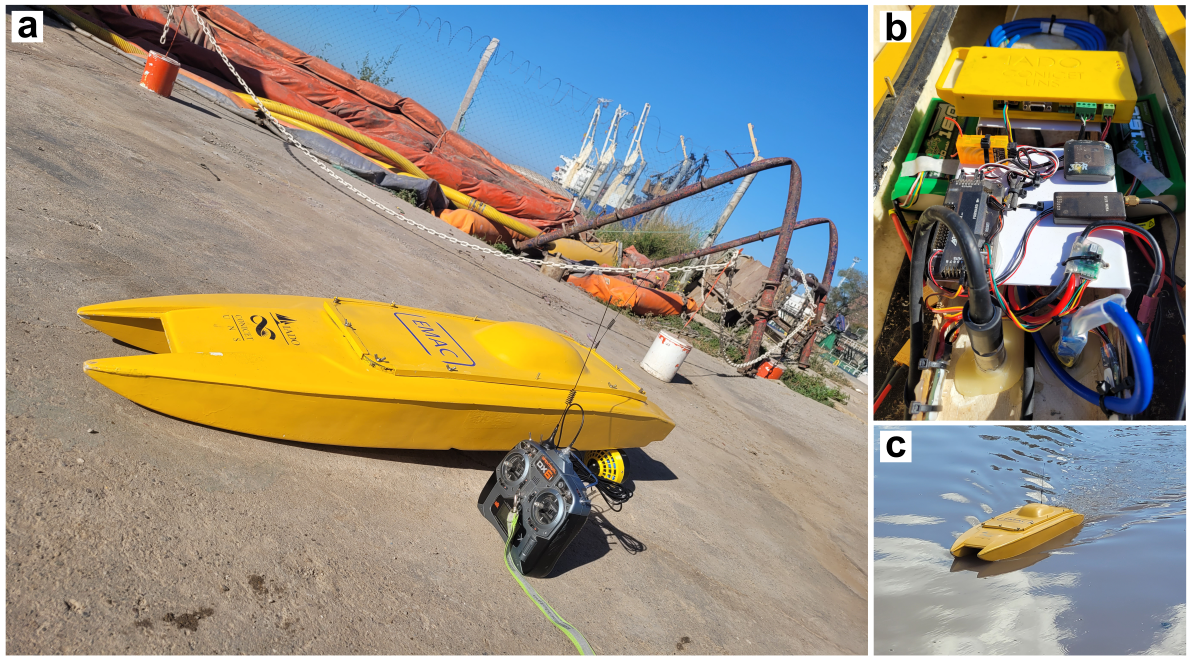

Supplement: Supplementary Data 1 [file mmc1.zip › MONITORING MULTIPLE PARAMETERS IN COMPLEX WATER SCENARIOS USING A LOW COST OPEN SOURCE DATA ACQUISITION PLATFORM/EMAC-USV working/EMAC-USV-TuzxMM.png]

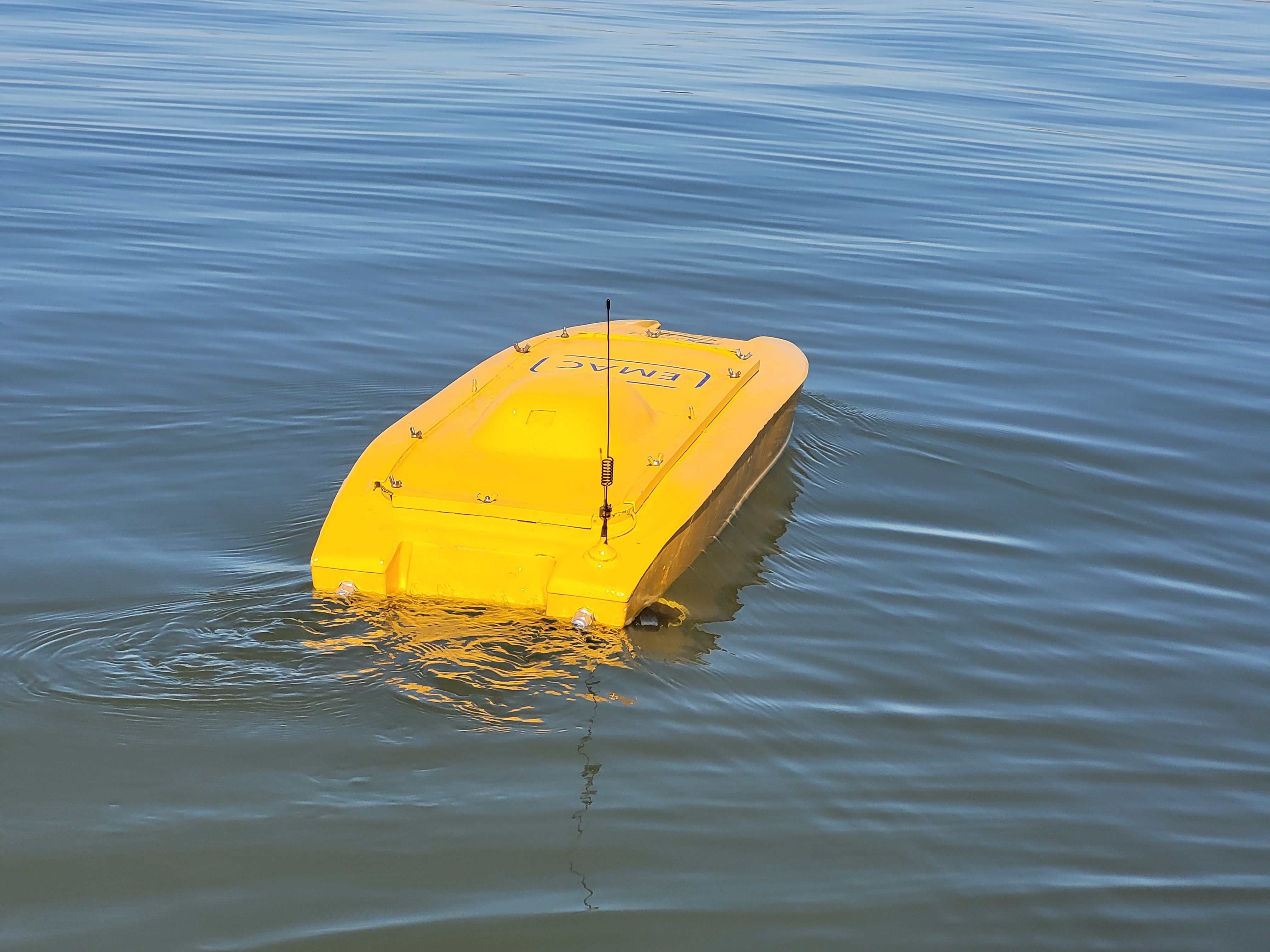

Supplement: Supplementary Data 1 [file mmc1.zip › MONITORING MULTIPLE PARAMETERS IN COMPLEX WATER SCENARIOS USING A LOW COST OPEN SOURCE DATA ACQUISITION PLATFORM/EMAC-USV working/20230505_123220.jpg]

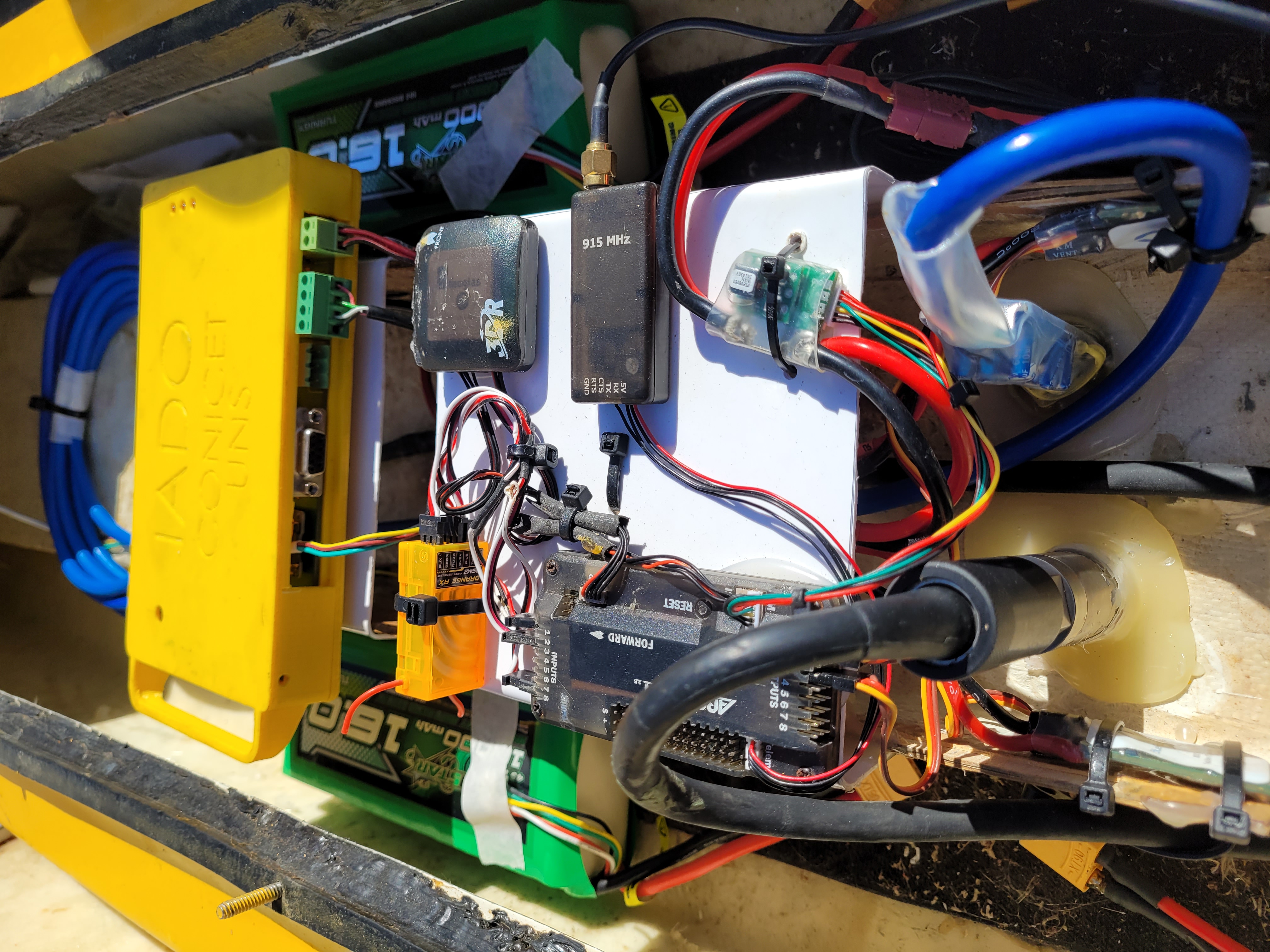

Supplement: Supplementary Data 1 [file mmc1.zip › MONITORING MULTIPLE PARAMETERS IN COMPLEX WATER SCENARIOS USING A LOW COST OPEN SOURCE DATA ACQUISITION PLATFORM/EMAC-USV working/20230505_122407.jpg]

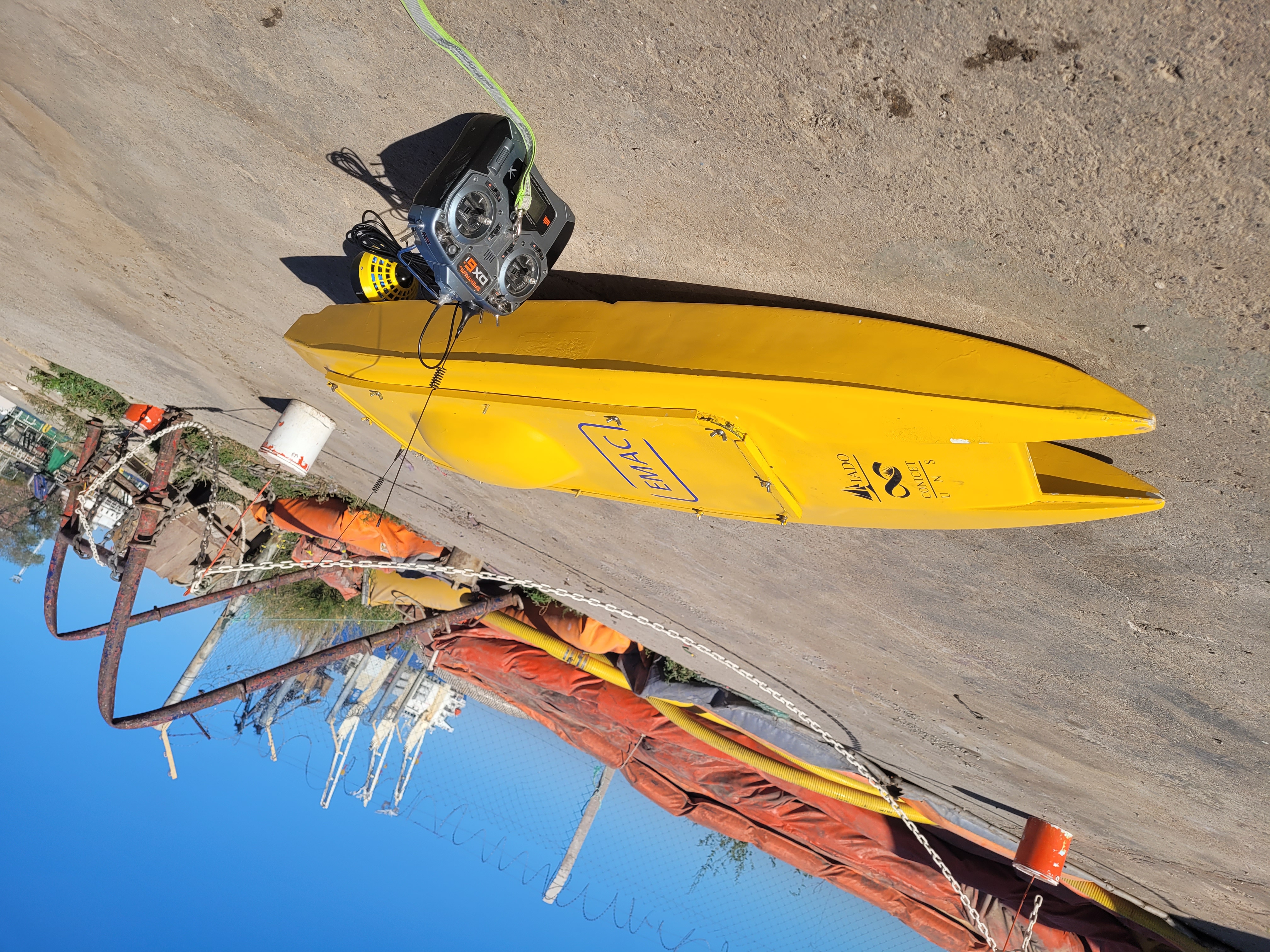

Supplement: Supplementary Data 1 [file mmc1.zip › MONITORING MULTIPLE PARAMETERS IN COMPLEX WATER SCENARIOS USING A LOW COST OPEN SOURCE DATA ACQUISITION PLATFORM/EMAC-USV working/20230505_112637.jpg]

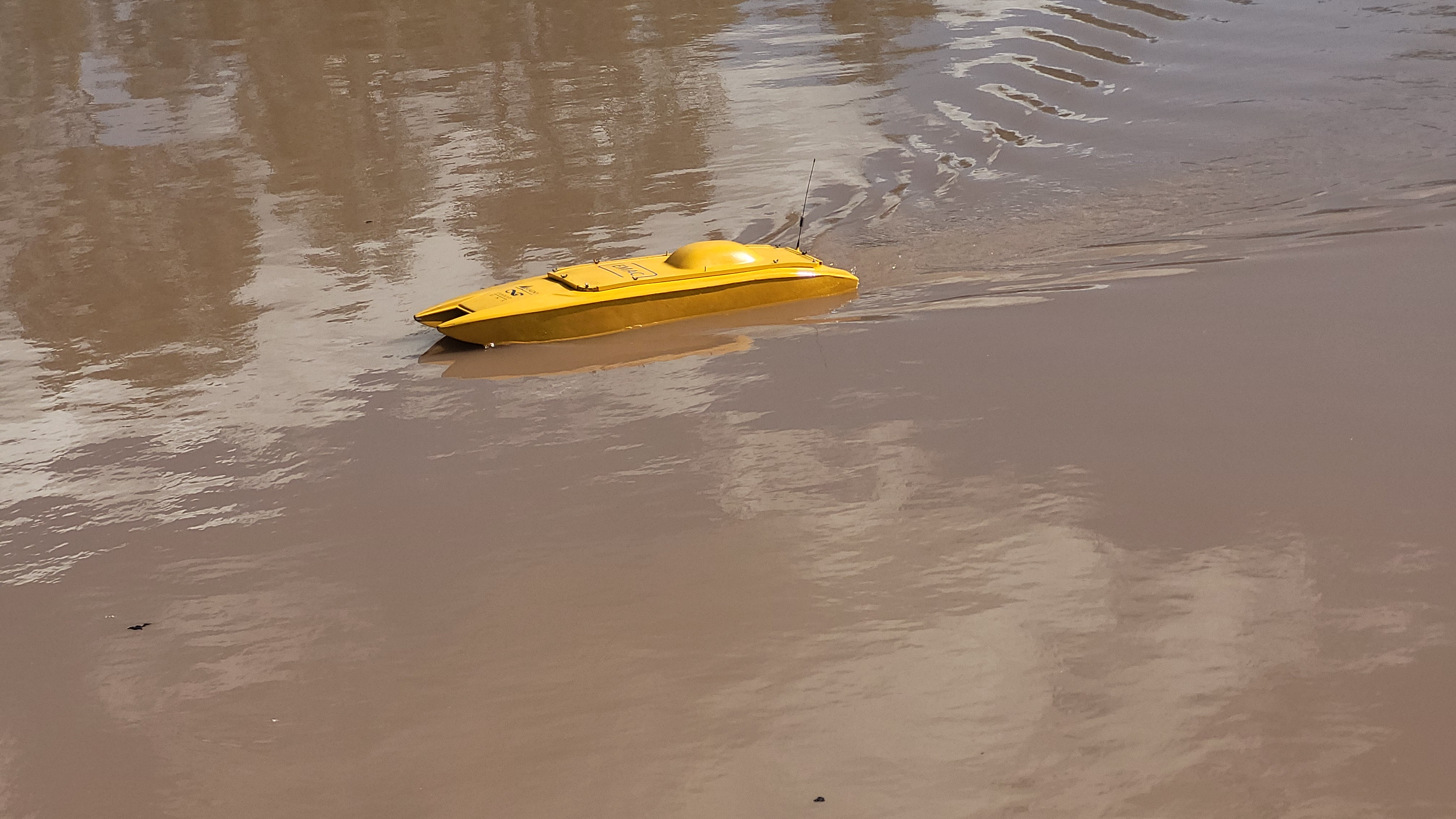

Supplement: Supplementary Data 1 [file mmc1.zip › MONITORING MULTIPLE PARAMETERS IN COMPLEX WATER SCENARIOS USING A LOW COST OPEN SOURCE DATA ACQUISITION PLATFORM/EMAC-USV working/20221026_160612.jpg]

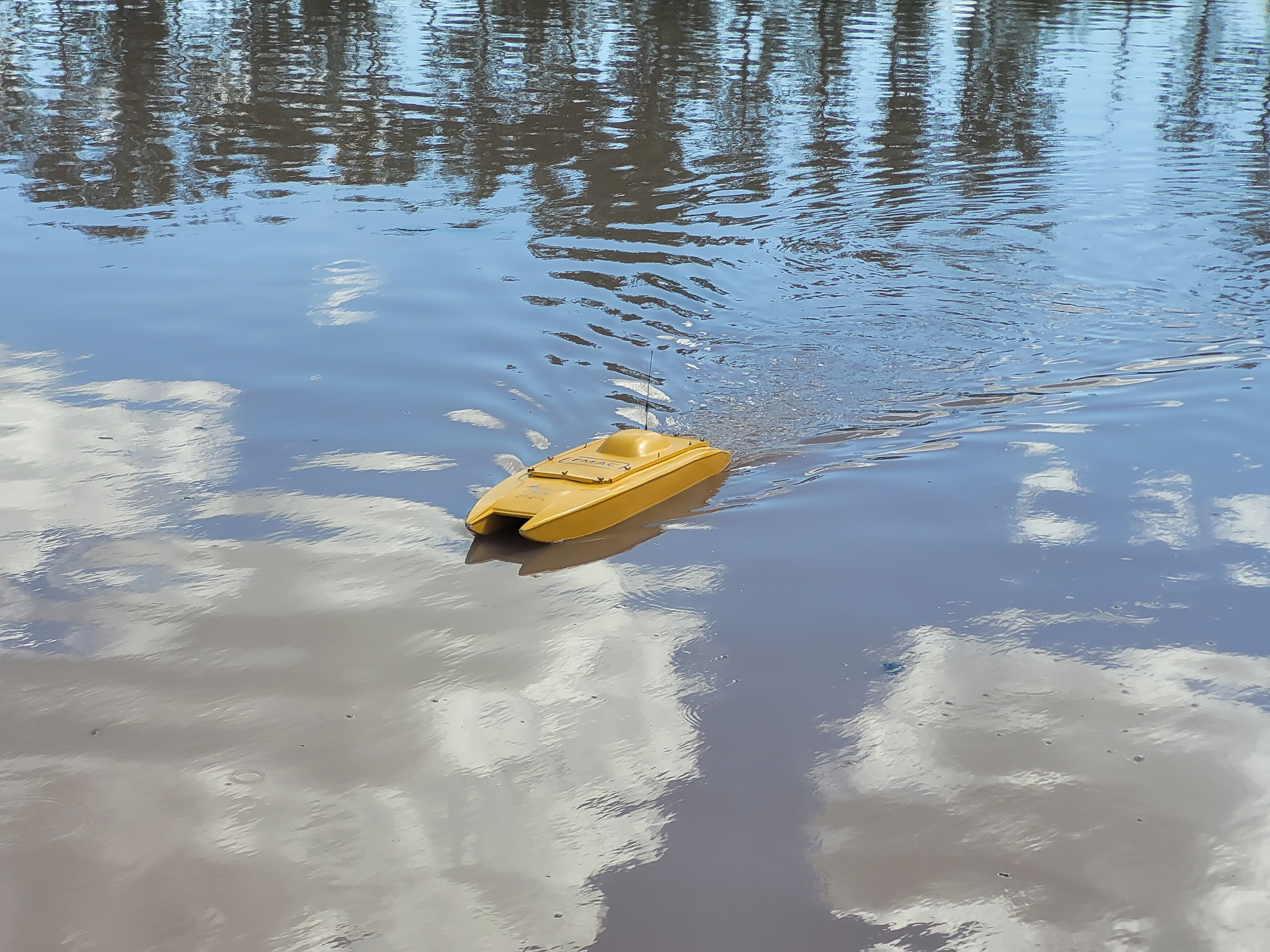

Supplement: Supplementary Data 1 [file mmc1.zip › MONITORING MULTIPLE PARAMETERS IN COMPLEX WATER SCENARIOS USING A LOW COST OPEN SOURCE DATA ACQUISITION PLATFORM/EMAC-USV working/20221026_160249.jpg]

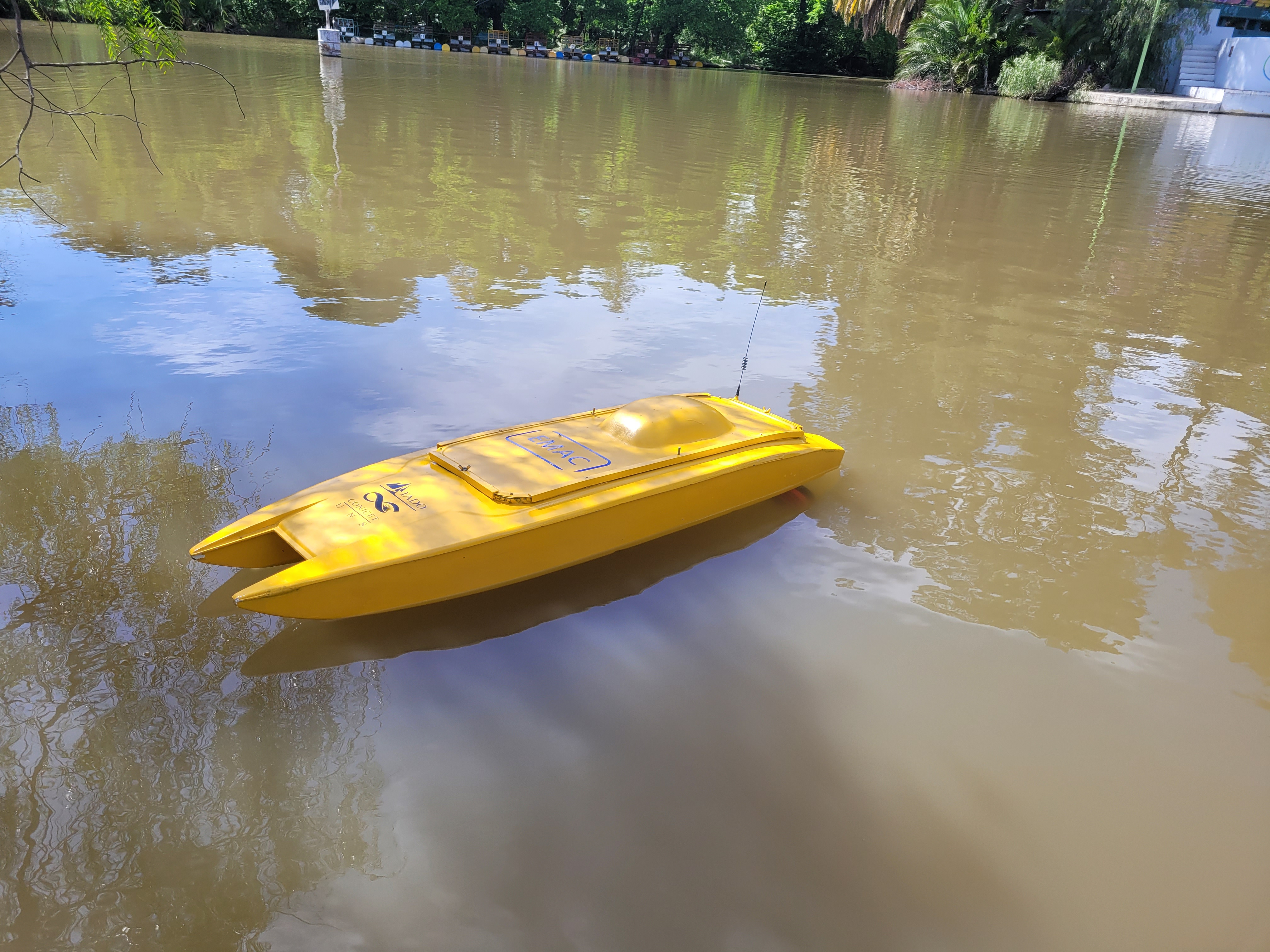

Supplement: Supplementary Data 1 [file mmc1.zip › MONITORING MULTIPLE PARAMETERS IN COMPLEX WATER SCENARIOS USING A LOW COST OPEN SOURCE DATA ACQUISITION PLATFORM/EMAC-USV working/20221024_105326.jpg]

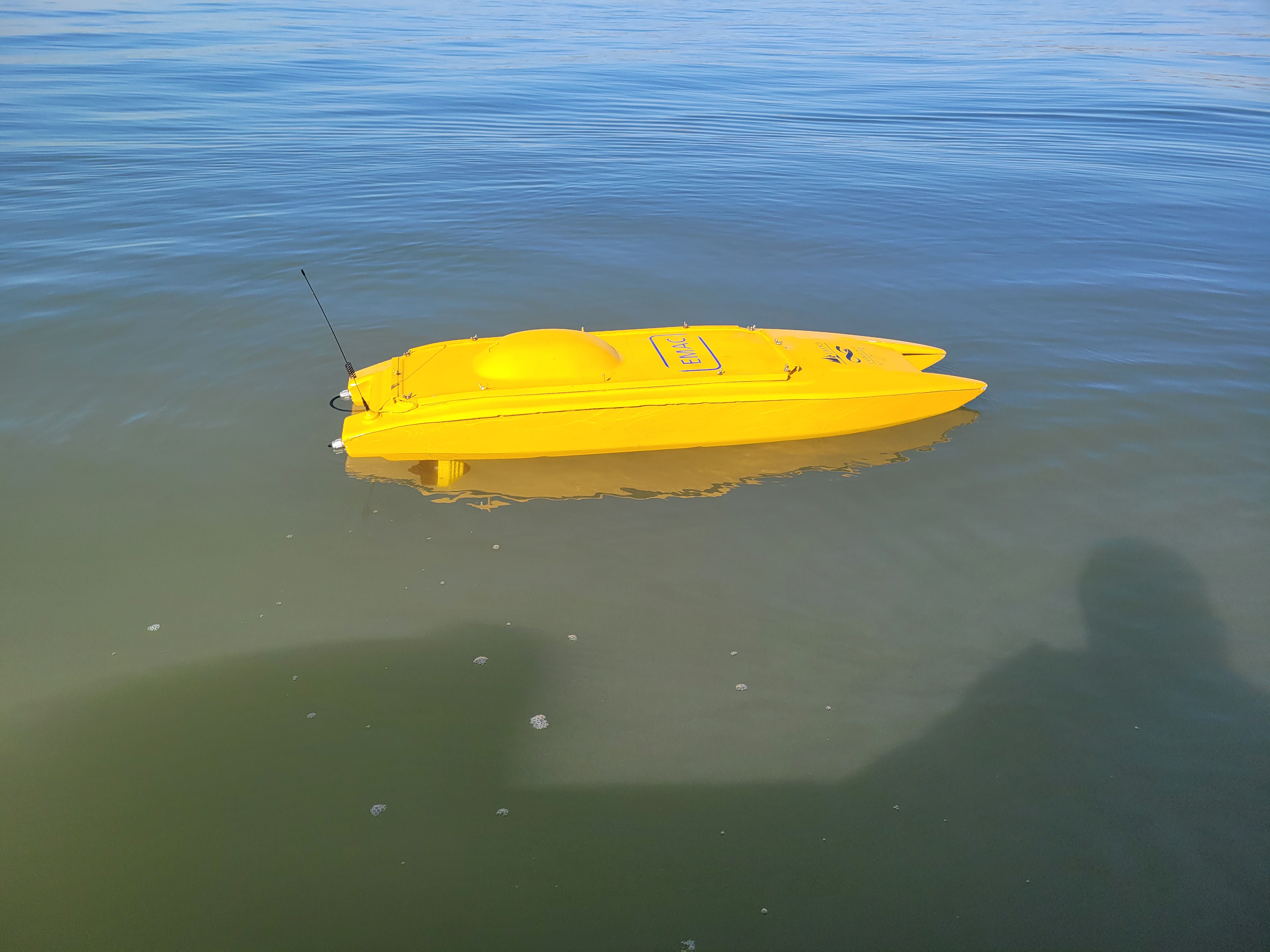

Supplement: Supplementary Data 1 [file mmc1.zip › MONITORING MULTIPLE PARAMETERS IN COMPLEX WATER SCENARIOS USING A LOW COST OPEN SOURCE DATA ACQUISITION PLATFORM/EMAC-USV working/20230505_123153.jpg]

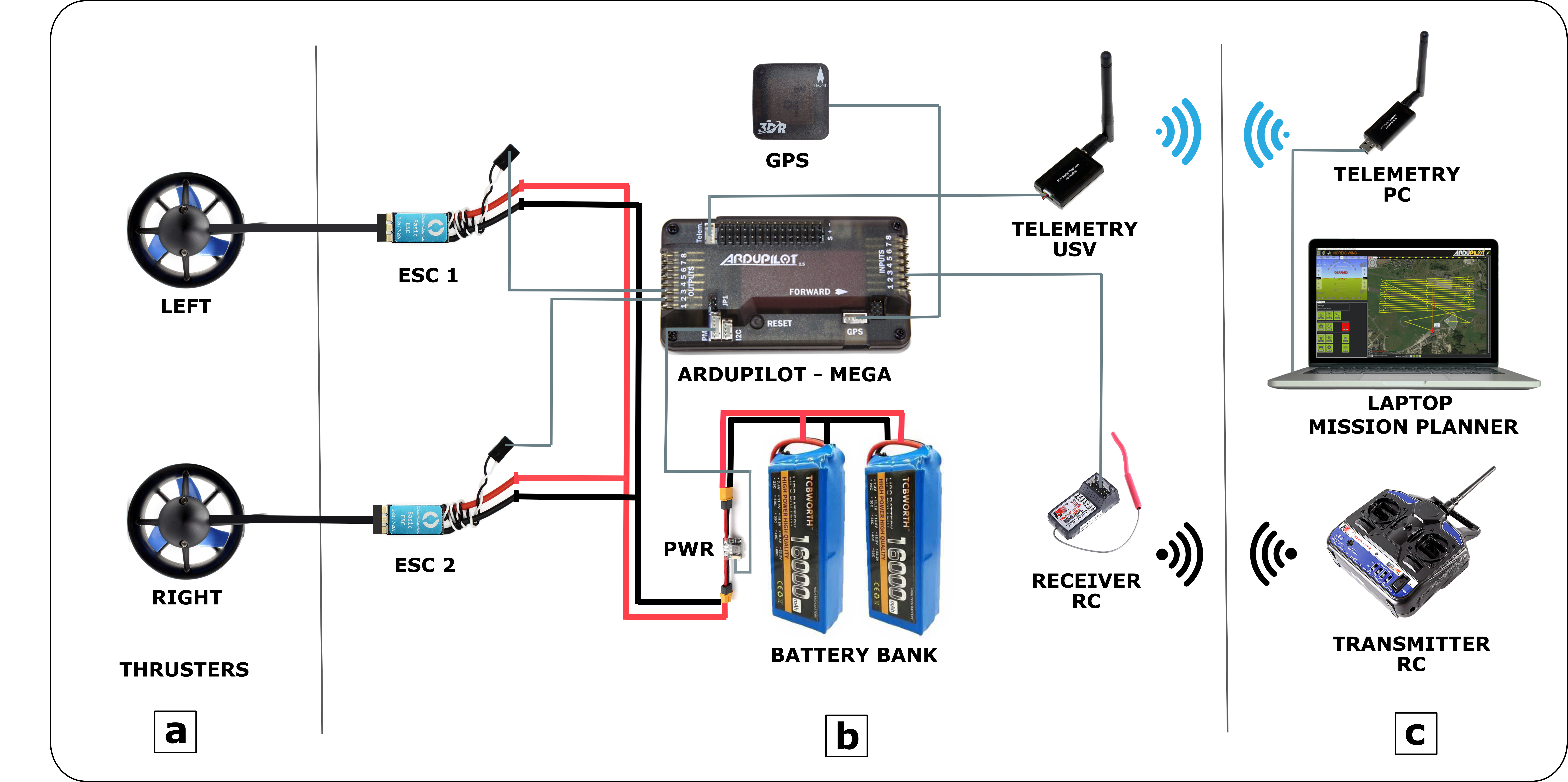

Supplement: Supplementary Data 1 [file mmc1.zip › MONITORING MULTIPLE PARAMETERS IN COMPLEX WATER SCENARIOS USING A LOW COST OPEN SOURCE DATA ACQUISITION PLATFORM/Hardware files/EMAC-USV architecture /Ardupilot-system.png]

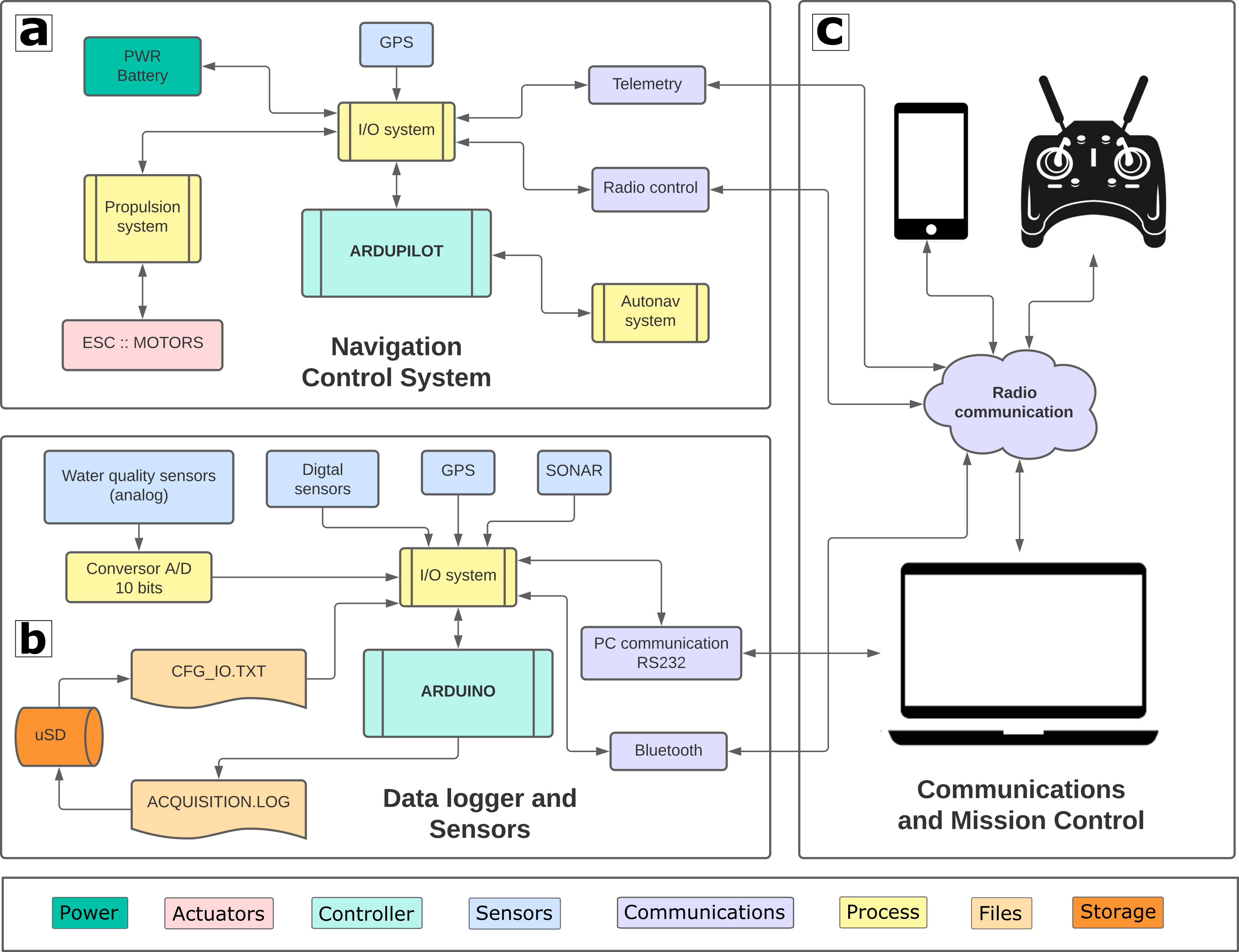

Supplement: Supplementary Data 1 [file mmc1.zip › MONITORING MULTIPLE PARAMETERS IN COMPLEX WATER SCENARIOS USING A LOW COST OPEN SOURCE DATA ACQUISITION PLATFORM/Hardware files/EMAC-USV architecture /EMAC-architecture.png]

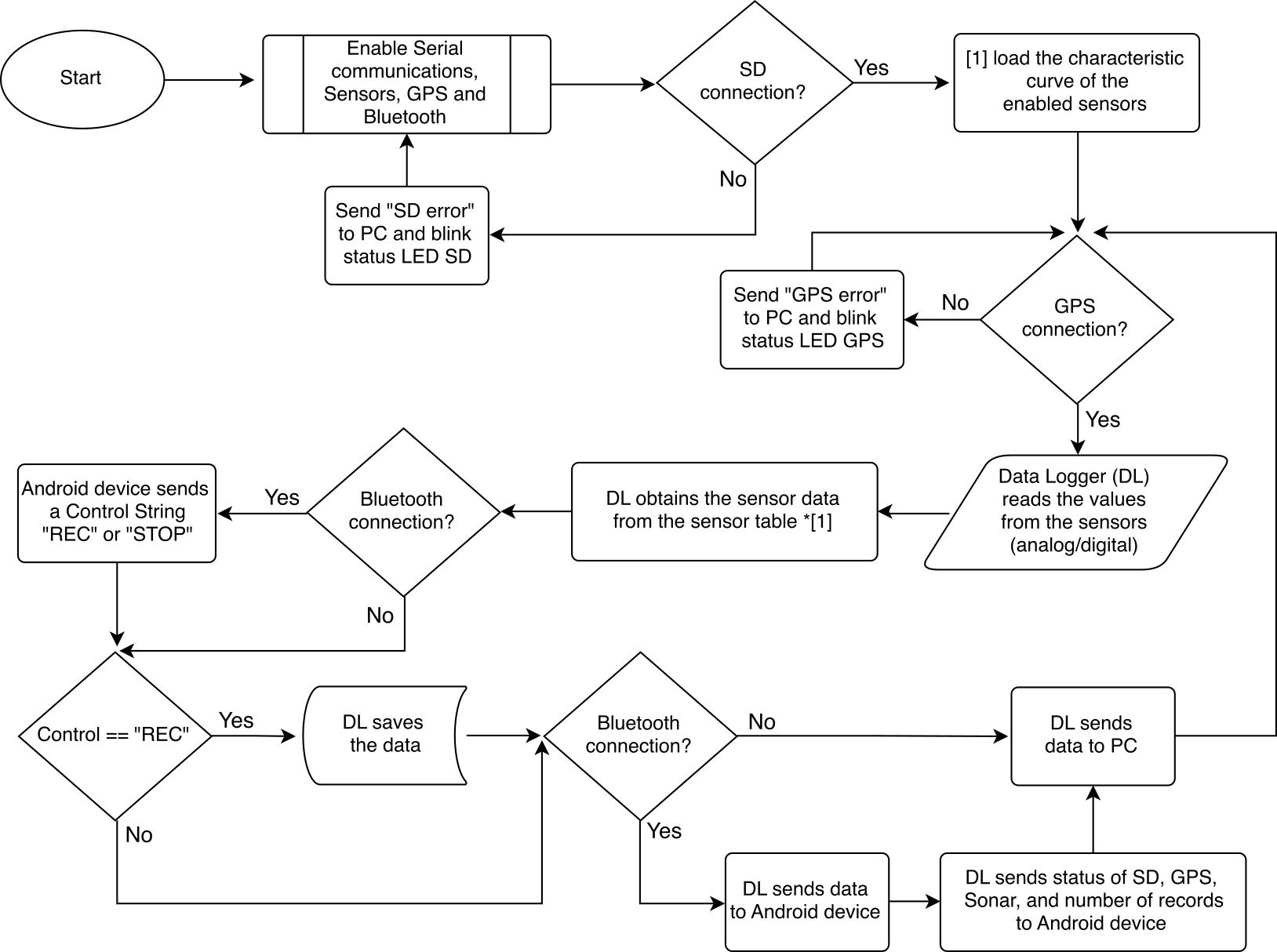

Supplement: Supplementary Data 1 [file mmc1.zip › MONITORING MULTIPLE PARAMETERS IN COMPLEX WATER SCENARIOS USING A LOW COST OPEN SOURCE DATA ACQUISITION PLATFORM/Software files/Data logger/Datalogger-Diagram.png]

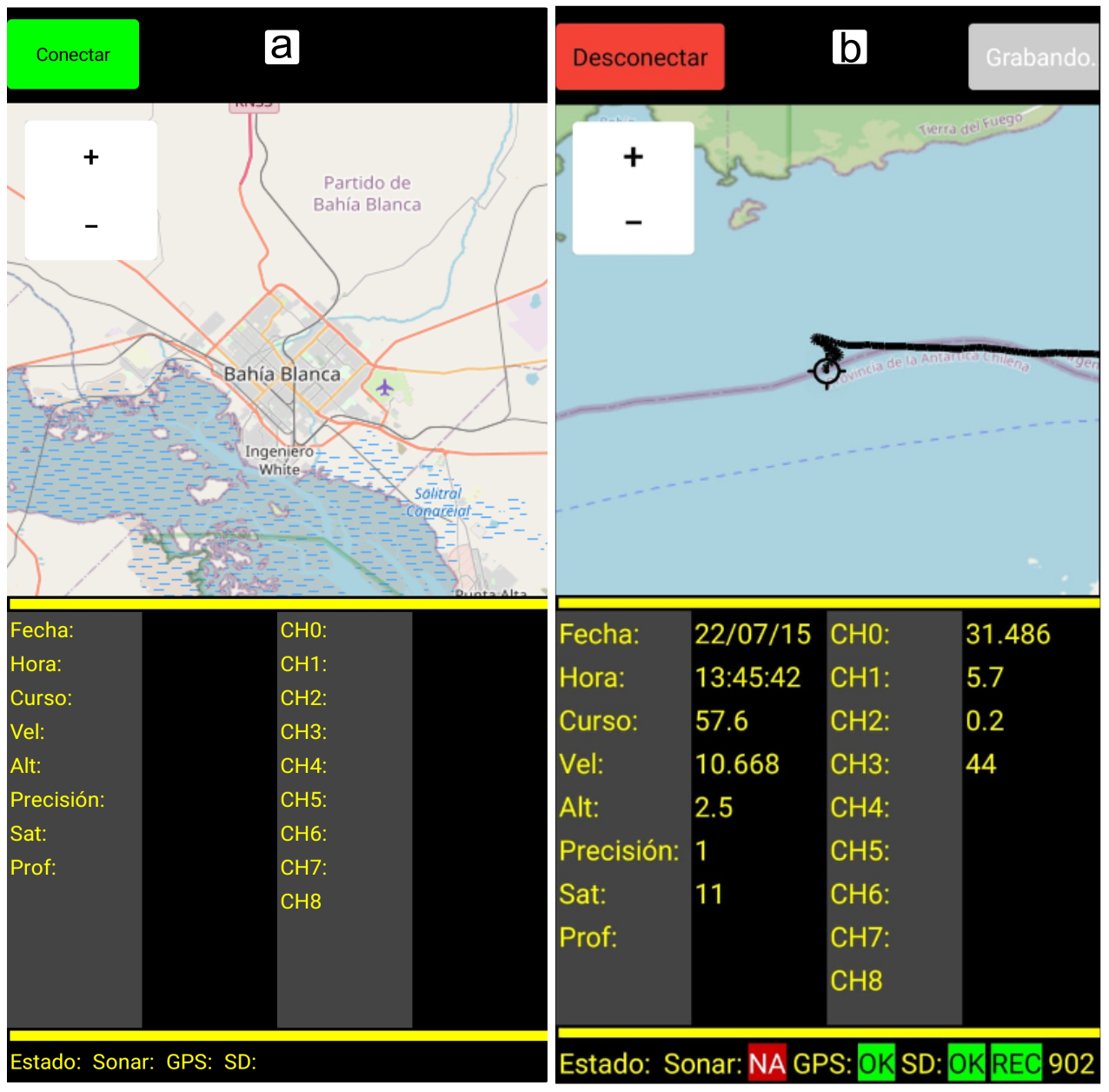

Supplement: Supplementary Data 1 [file mmc1.zip › MONITORING MULTIPLE PARAMETERS IN COMPLEX WATER SCENARIOS USING A LOW COST OPEN SOURCE DATA ACQUISITION PLATFORM/Software files/Mobile app/Dashboard.png]

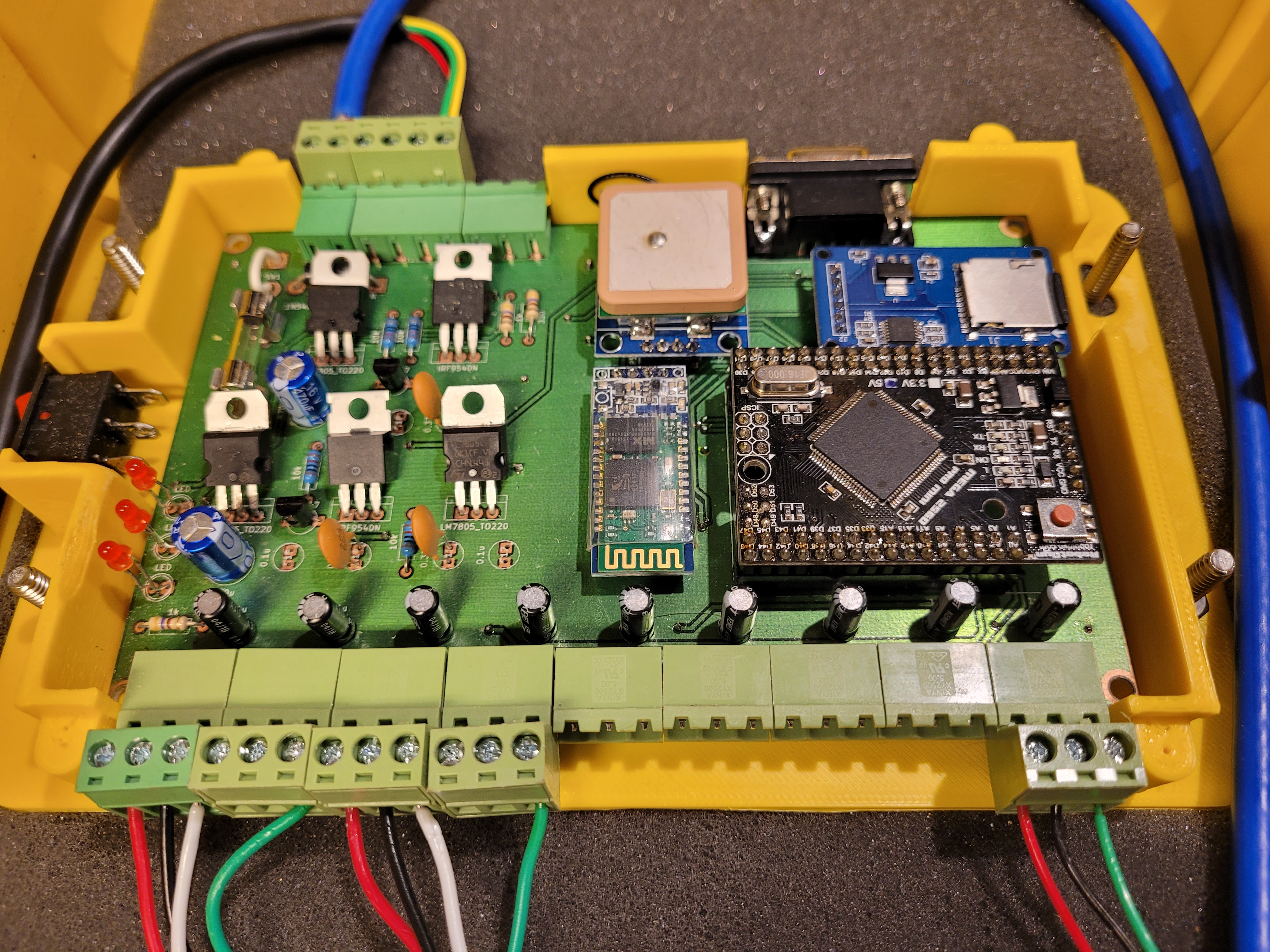

Supplement: Supplementary Data 1 [file mmc1.zip › MONITORING MULTIPLE PARAMETERS IN COMPLEX WATER SCENARIOS USING A LOW COST OPEN SOURCE DATA ACQUISITION PLATFORM/Hardware files/Data logger/Images/Datalogger_2.jpg]

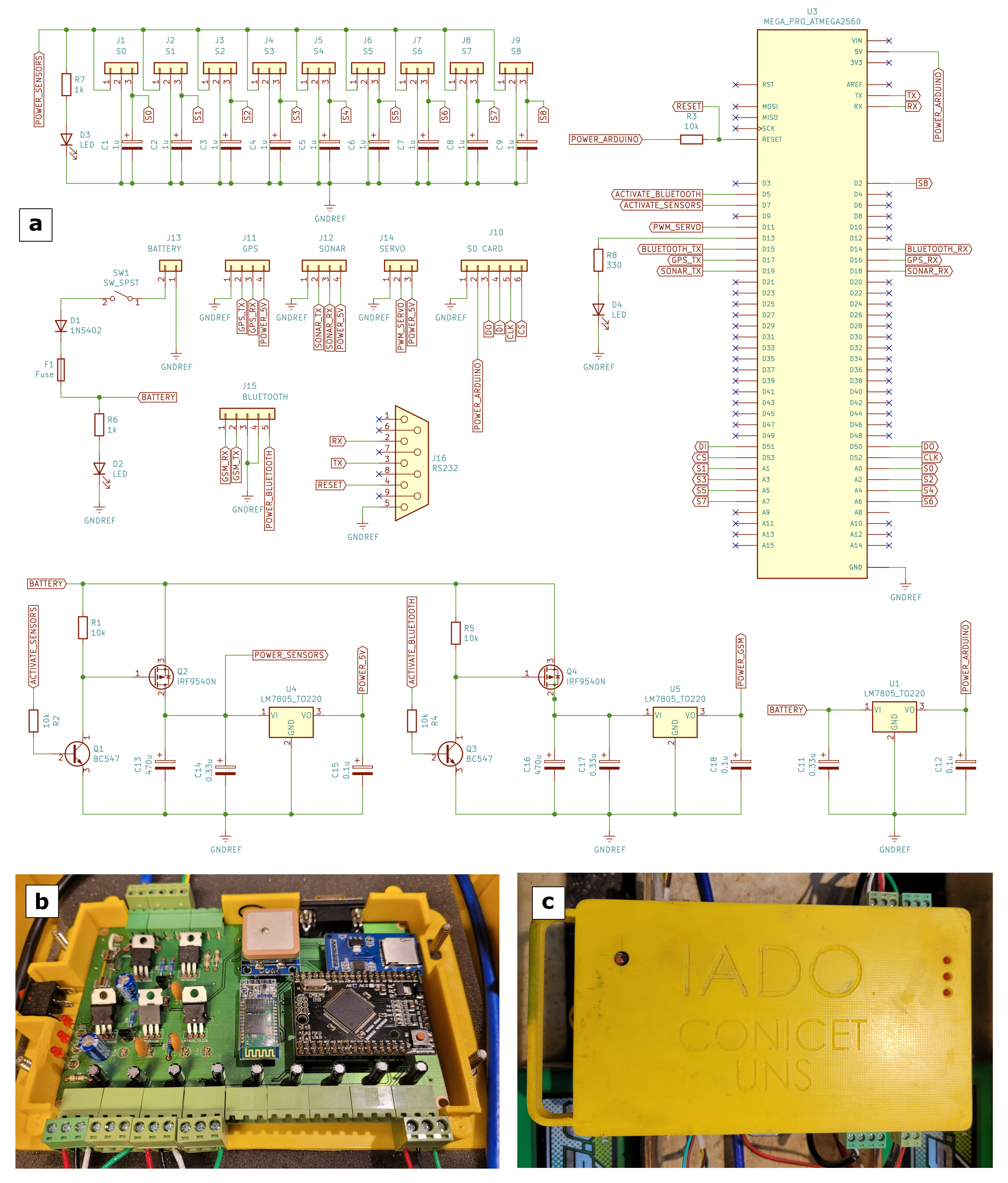

Supplement: Supplementary Data 1 [file mmc1.zip › MONITORING MULTIPLE PARAMETERS IN COMPLEX WATER SCENARIOS USING A LOW COST OPEN SOURCE DATA ACQUISITION PLATFORM/Hardware files/Data logger/Images/Datalogger-schematic.png]

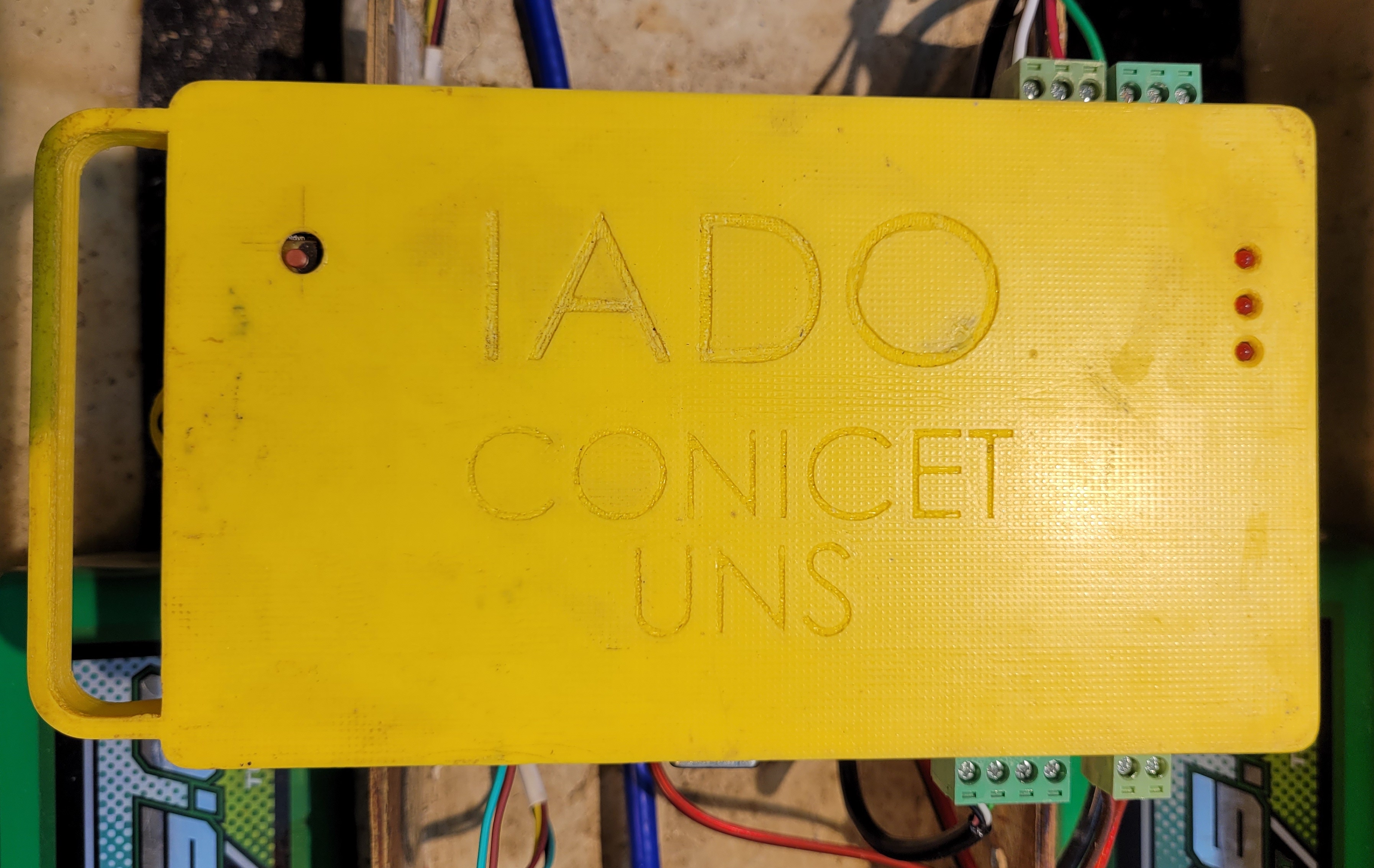

Supplement: Supplementary Data 1 [file mmc1.zip › MONITORING MULTIPLE PARAMETERS IN COMPLEX WATER SCENARIOS USING A LOW COST OPEN SOURCE DATA ACQUISITION PLATFORM/Hardware files/Data logger/Images/Datalogger_1.jpg]

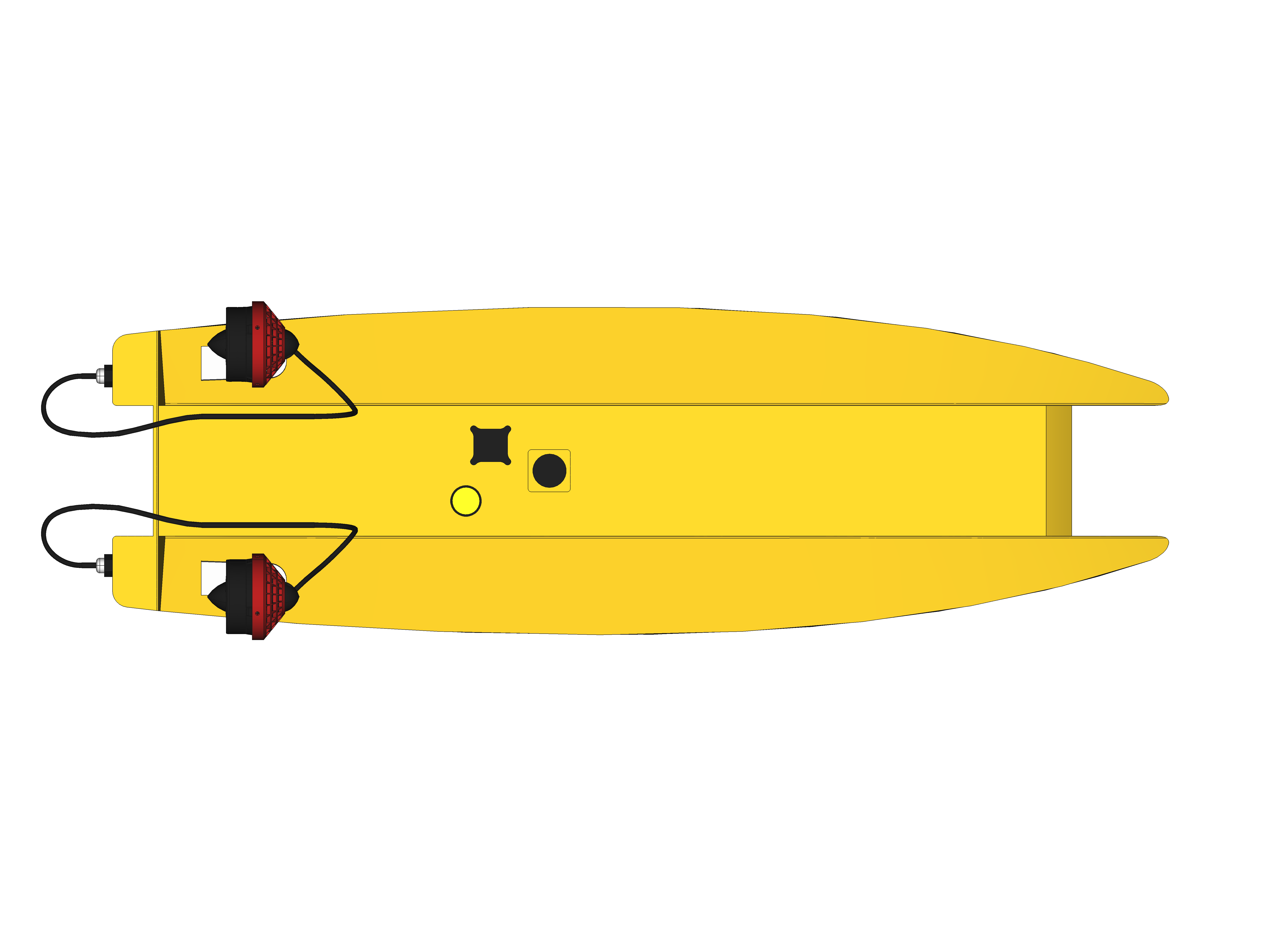

Supplement: Supplementary Data 1 [file mmc1.zip › MONITORING MULTIPLE PARAMETERS IN COMPLEX WATER SCENARIOS USING A LOW COST OPEN SOURCE DATA ACQUISITION PLATFORM/Hardware files/Hull/Images/USV3-XldXj2.png]

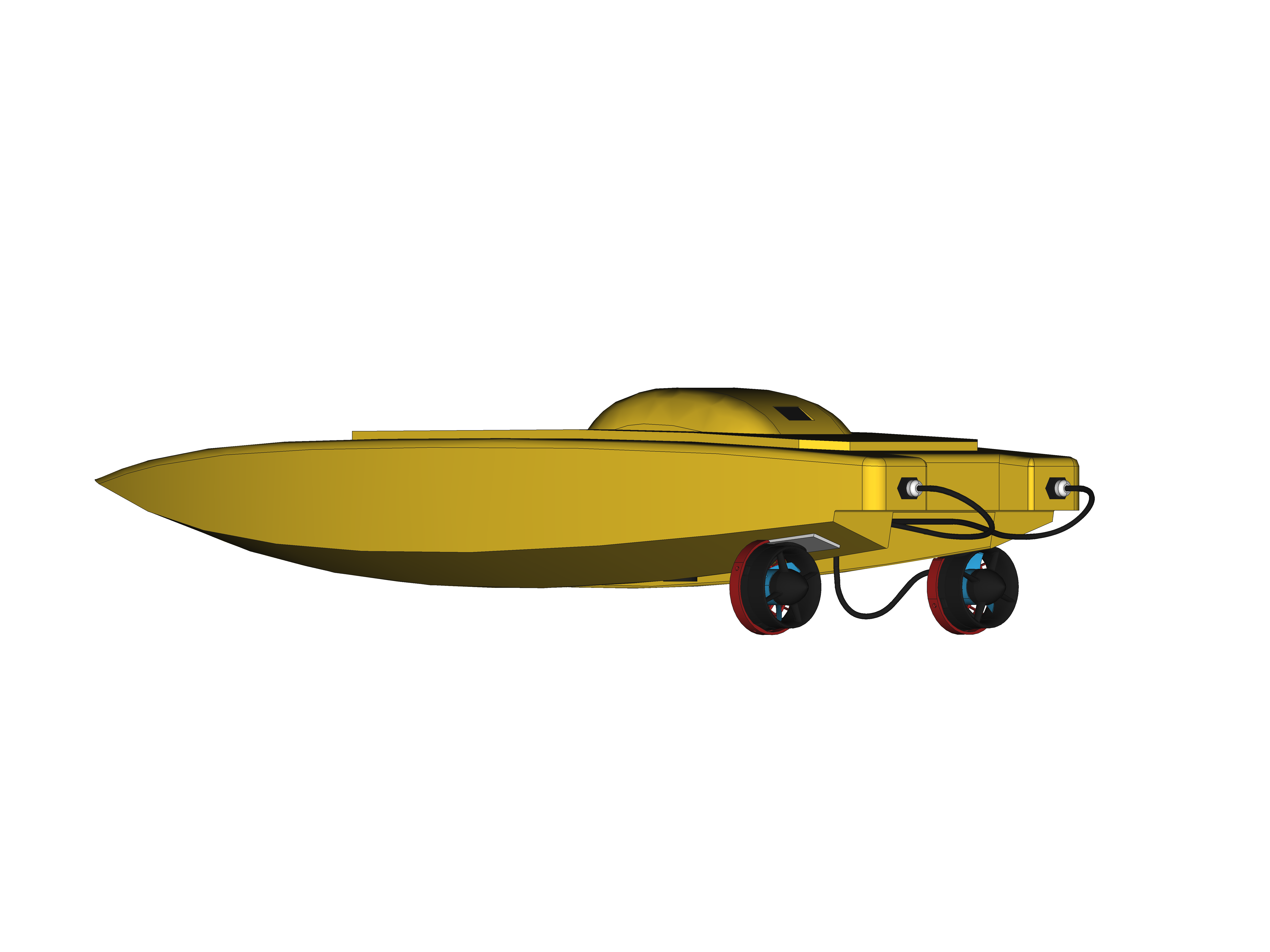

Supplement: Supplementary Data 1 [file mmc1.zip › MONITORING MULTIPLE PARAMETERS IN COMPLEX WATER SCENARIOS USING A LOW COST OPEN SOURCE DATA ACQUISITION PLATFORM/Hardware files/Hull/Images/USV2-k7bKsT.png]

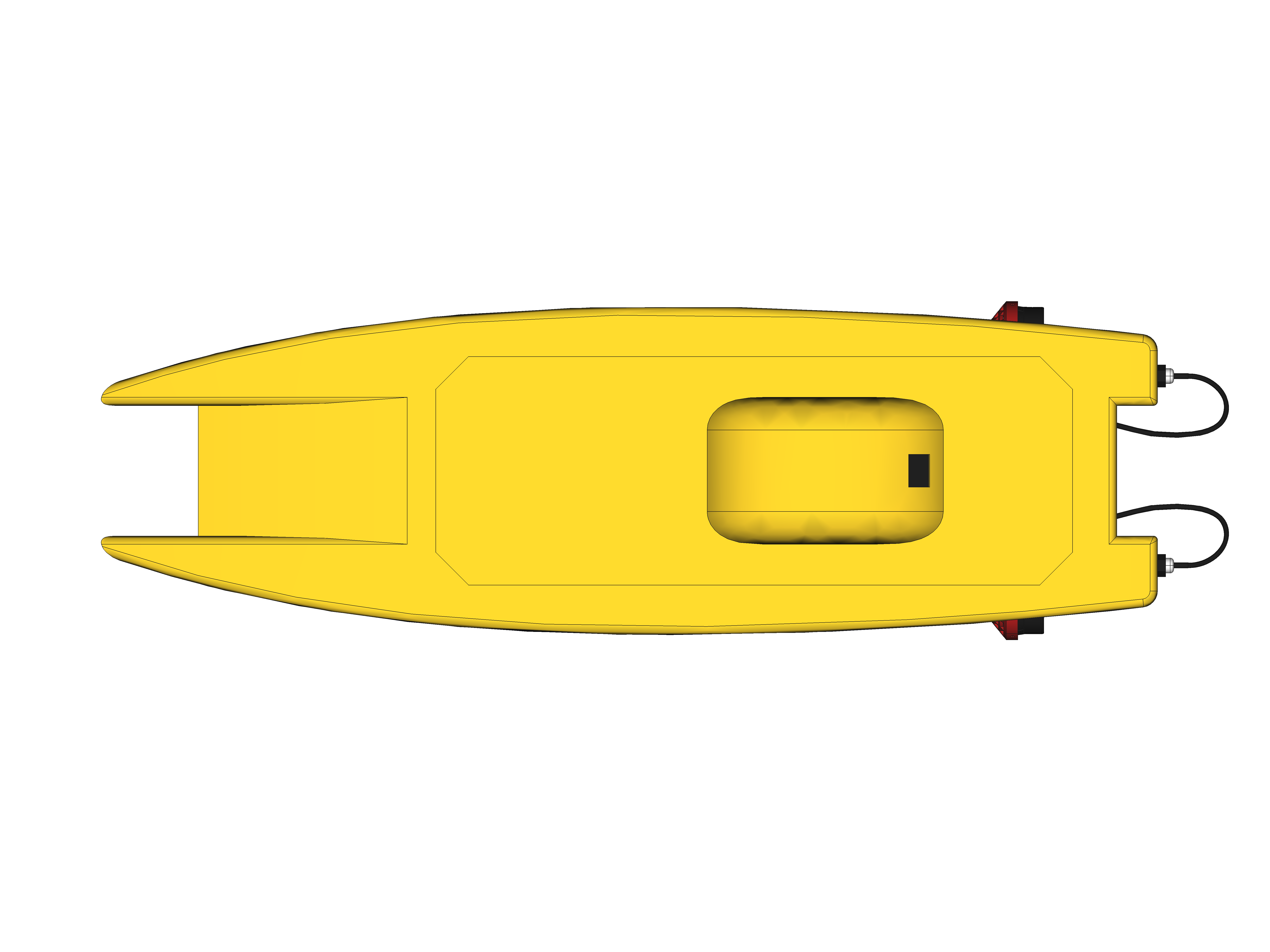

Supplement: Supplementary Data 1 [file mmc1.zip › MONITORING MULTIPLE PARAMETERS IN COMPLEX WATER SCENARIOS USING A LOW COST OPEN SOURCE DATA ACQUISITION PLATFORM/Hardware files/Hull/Images/USV4-5XaUd7.png]

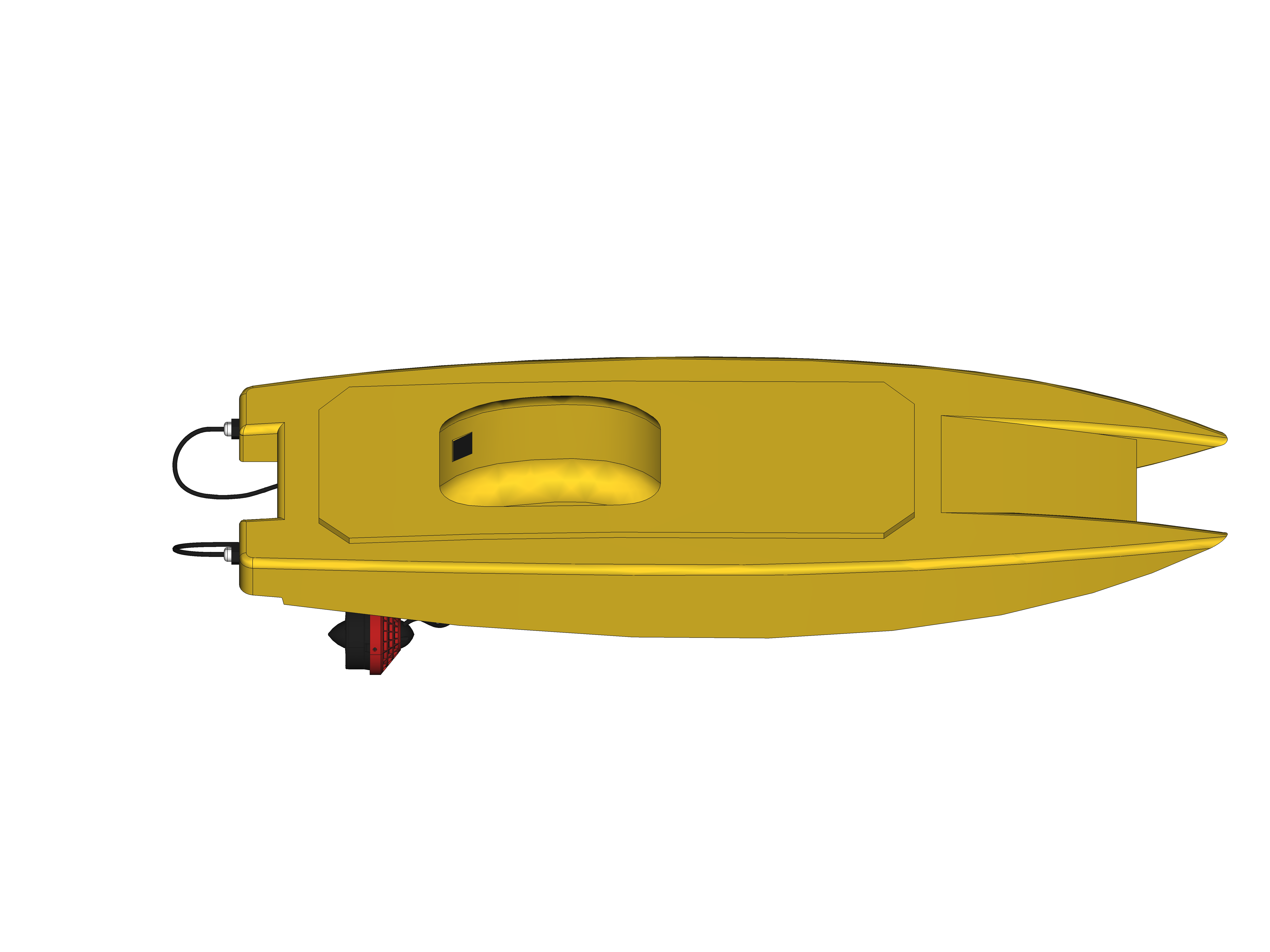

Supplement: Supplementary Data 1 [file mmc1.zip › MONITORING MULTIPLE PARAMETERS IN COMPLEX WATER SCENARIOS USING A LOW COST OPEN SOURCE DATA ACQUISITION PLATFORM/Hardware files/Hull/Images/USV1-q8q0cb.png]

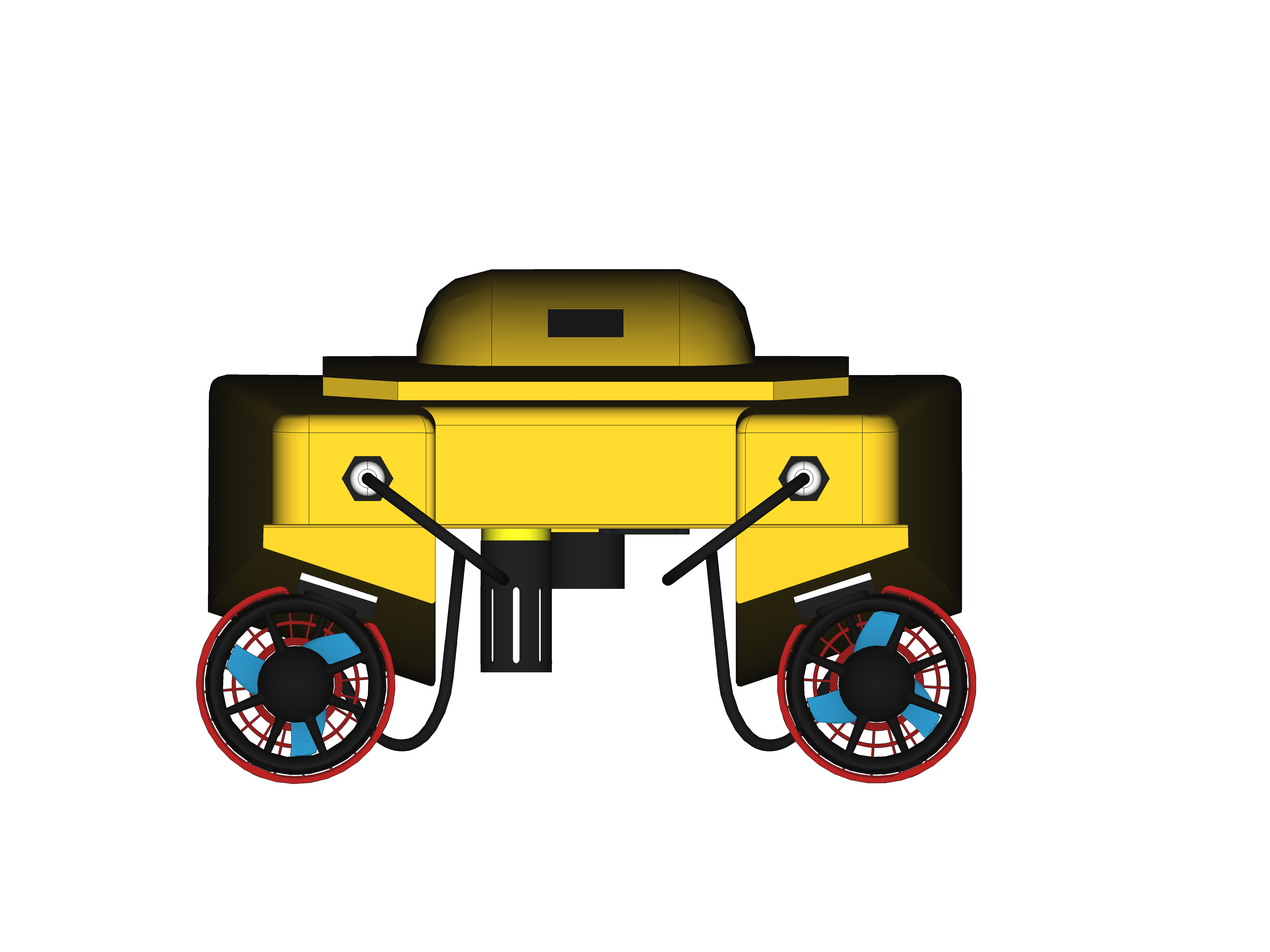

Supplement: Supplementary Data 1 [file mmc1.zip › MONITORING MULTIPLE PARAMETERS IN COMPLEX WATER SCENARIOS USING A LOW COST OPEN SOURCE DATA ACQUISITION PLATFORM/Hardware files/Hull/Images/USV5-Bhd1i8.png]

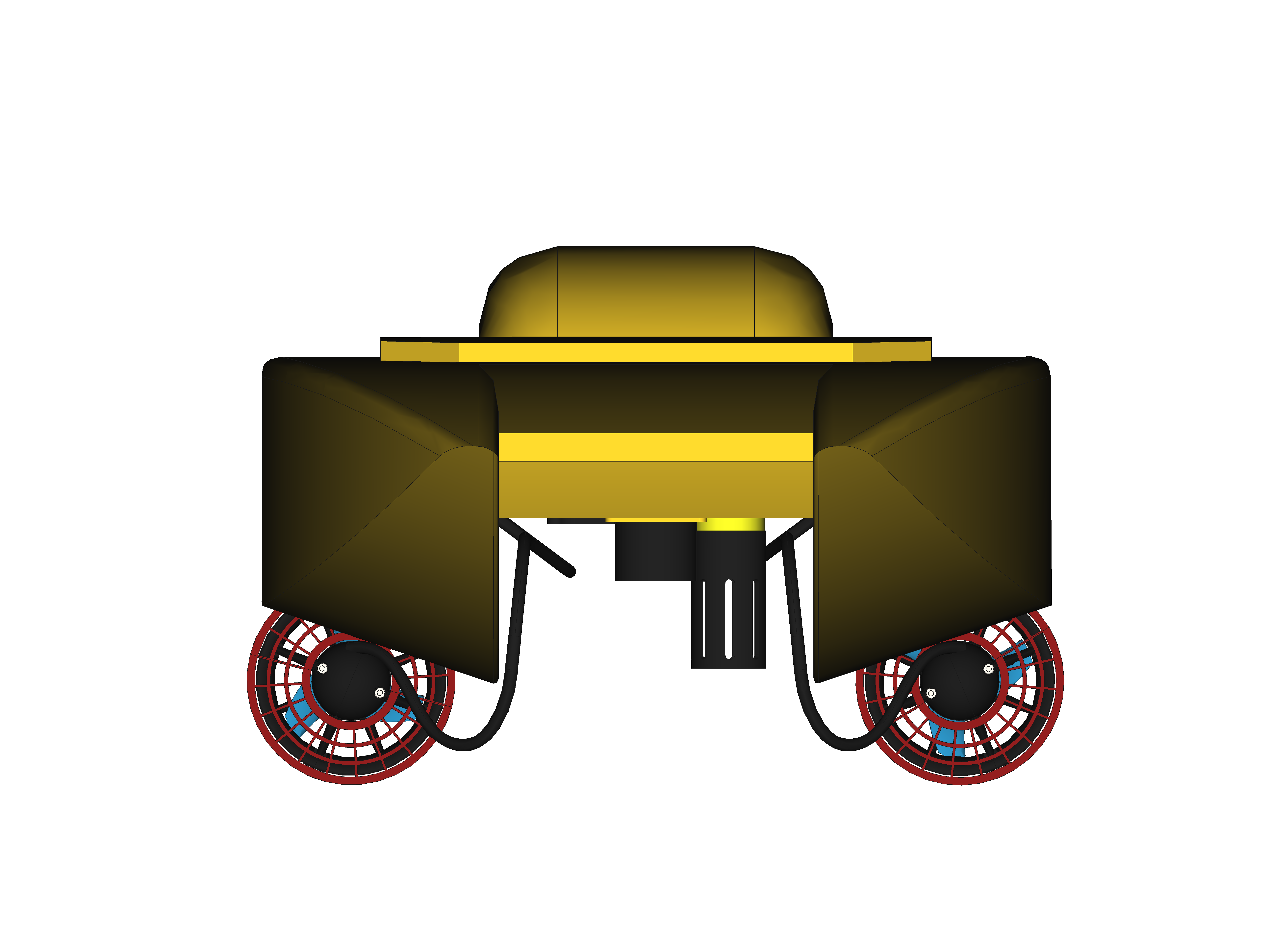

Supplement: Supplementary Data 1 [file mmc1.zip › MONITORING MULTIPLE PARAMETERS IN COMPLEX WATER SCENARIOS USING A LOW COST OPEN SOURCE DATA ACQUISITION PLATFORM/Hardware files/Hull/Images/USV6-5jCkpw.png]

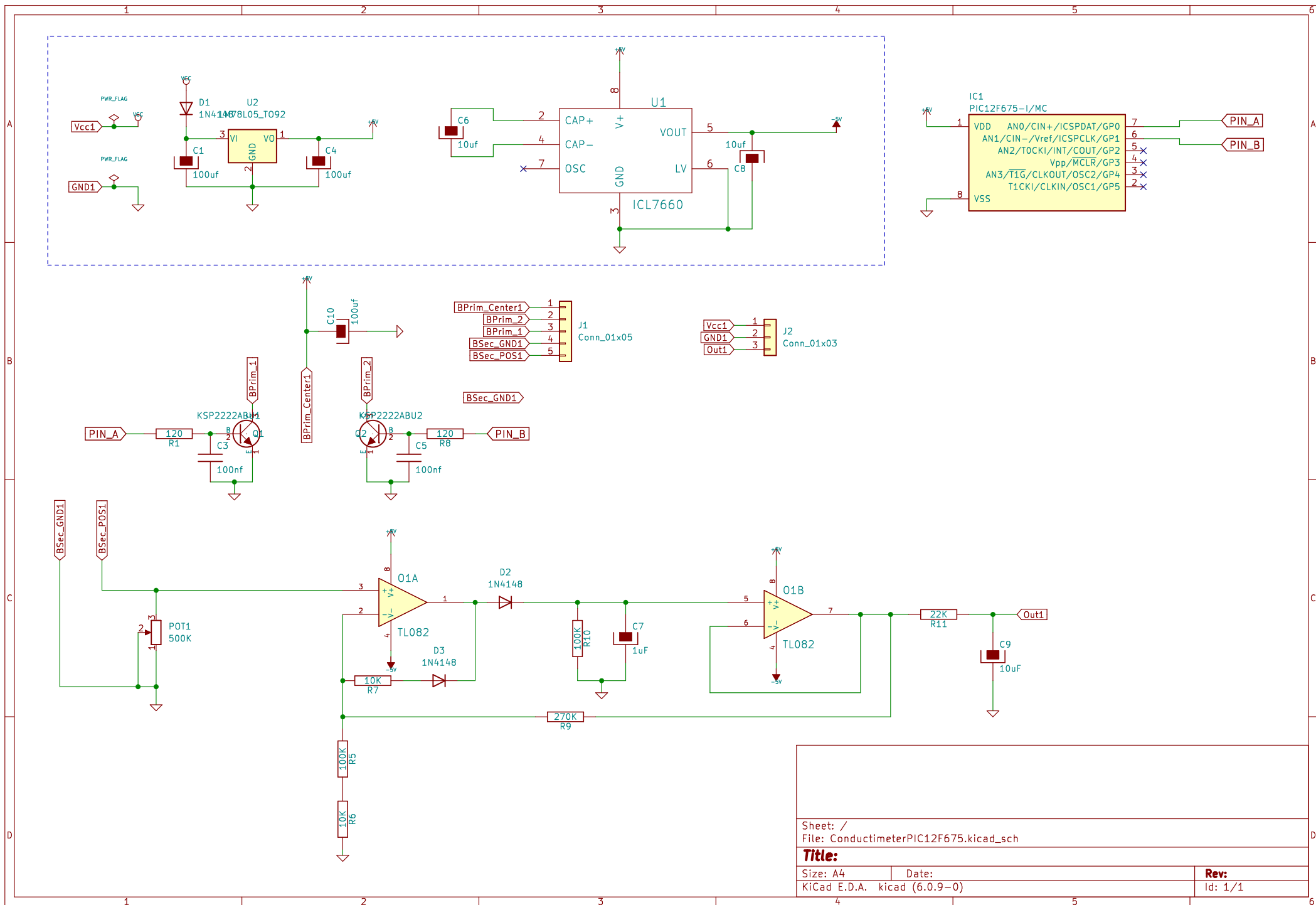

Supplement: Supplementary Data 1 [file mmc1.zip › MONITORING MULTIPLE PARAMETERS IN COMPLEX WATER SCENARIOS USING A LOW COST OPEN SOURCE DATA ACQUISITION PLATFORM/Hardware files/Sensors/Temperature/Temperature.pdf]

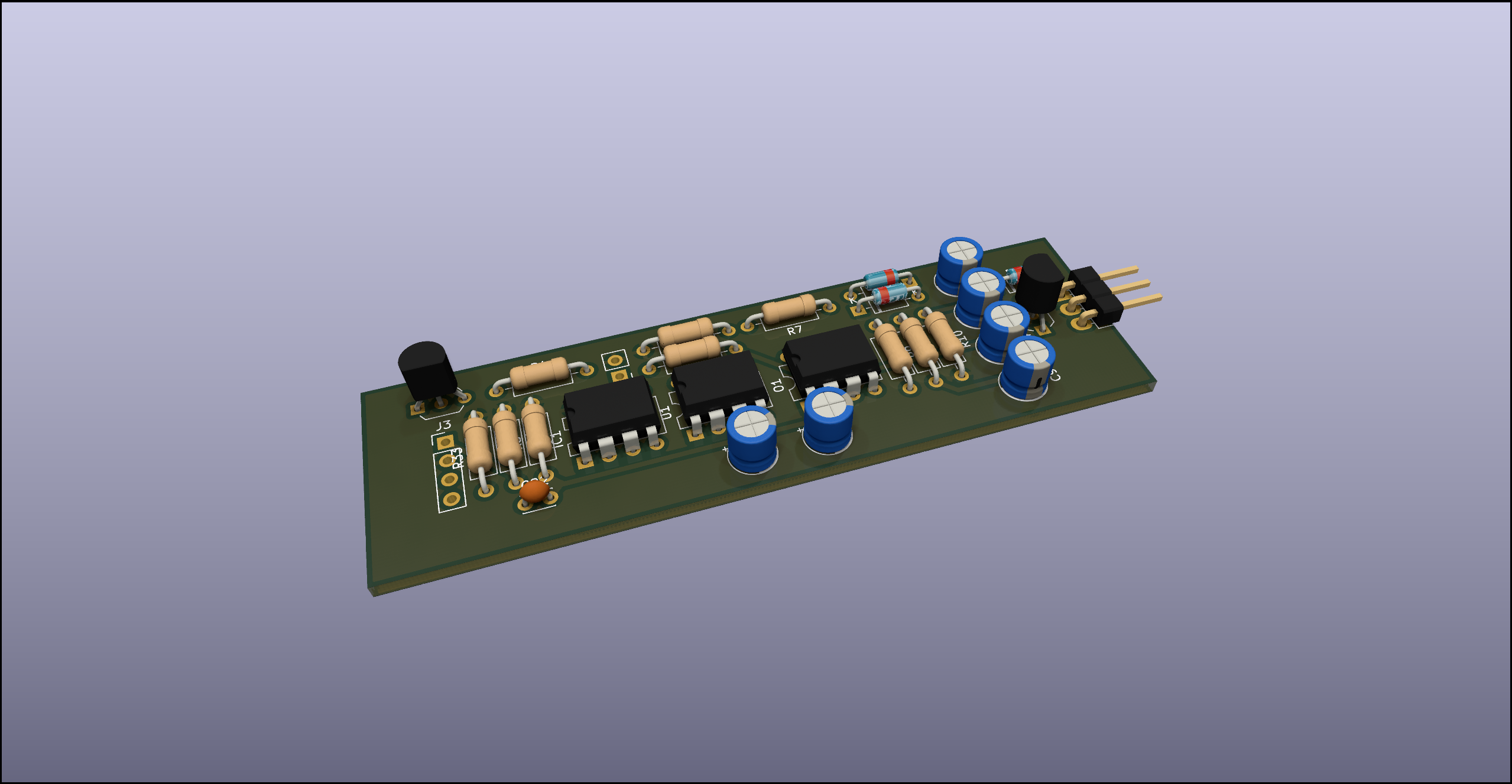

Supplement: Supplementary Data 1 [file mmc1.zip › MONITORING MULTIPLE PARAMETERS IN COMPLEX WATER SCENARIOS USING A LOW COST OPEN SOURCE DATA ACQUISITION PLATFORM/Hardware files/Sensors/Suspended solids concentration /Images/OBS_PIC12F675_00.png]

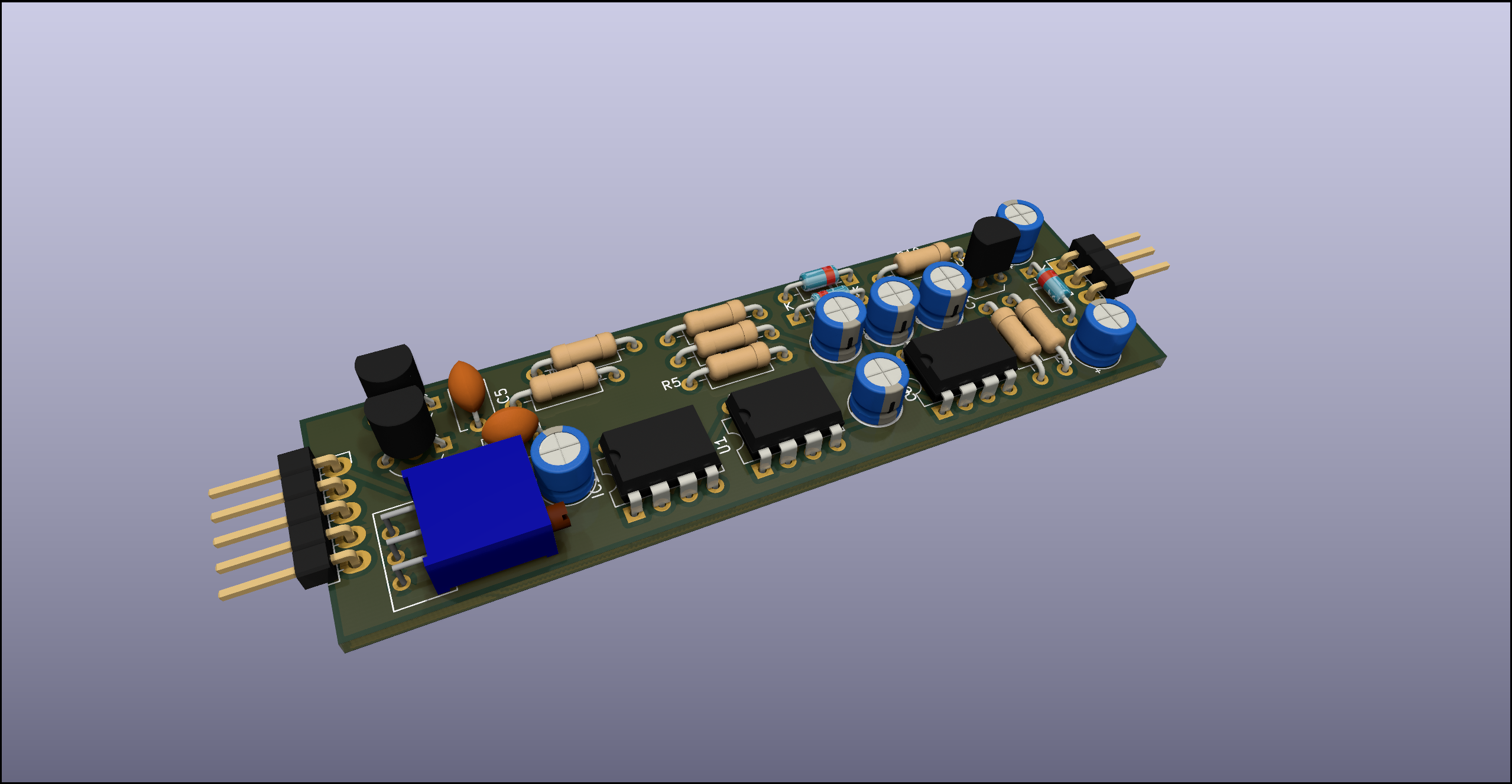

Supplement: Supplementary Data 1 [file mmc1.zip › MONITORING MULTIPLE PARAMETERS IN COMPLEX WATER SCENARIOS USING A LOW COST OPEN SOURCE DATA ACQUISITION PLATFORM/Hardware files/Sensors/Temperature/Images/ConductimeterPIC12F675_01.png]

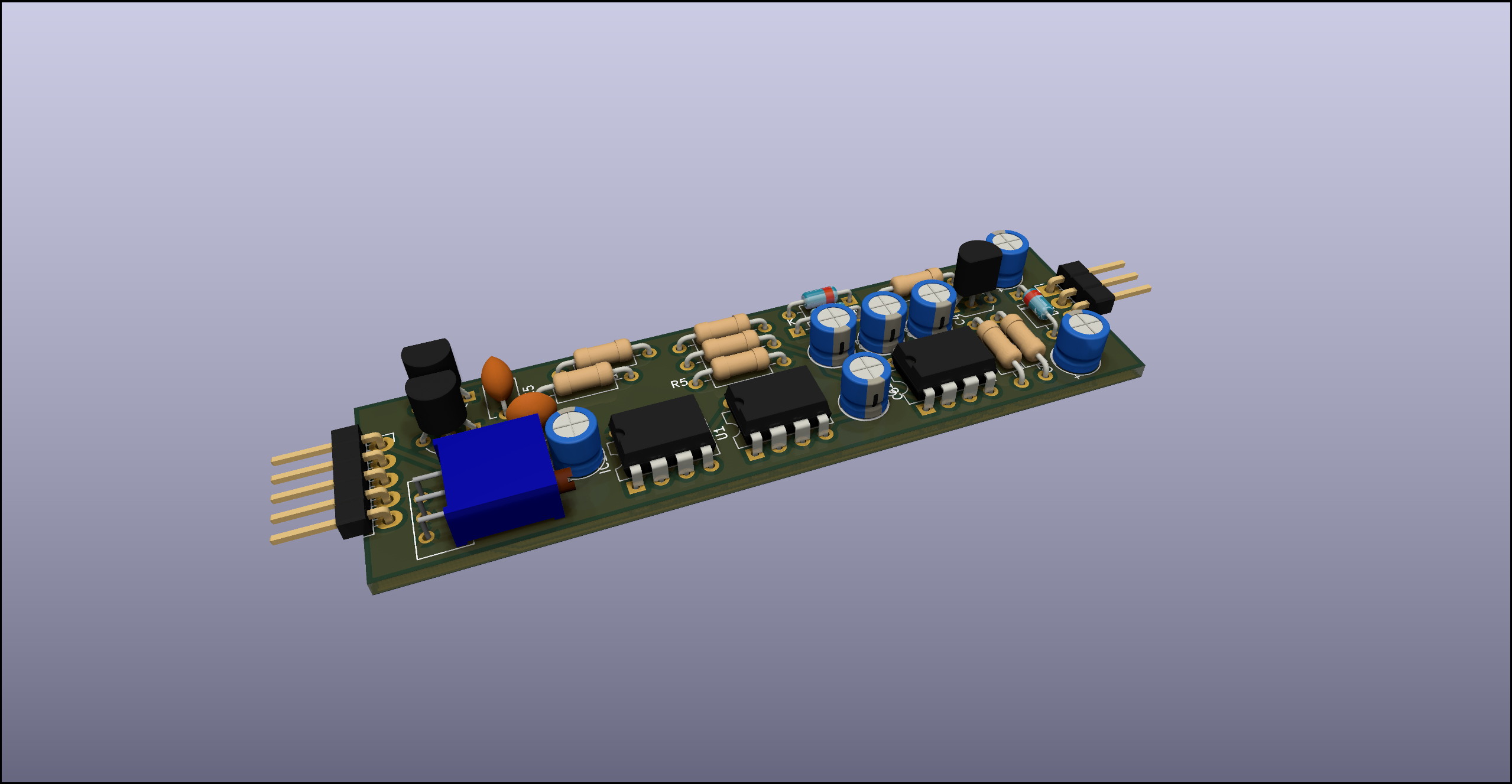

Supplement: Supplementary Data 1 [file mmc1.zip › MONITORING MULTIPLE PARAMETERS IN COMPLEX WATER SCENARIOS USING A LOW COST OPEN SOURCE DATA ACQUISITION PLATFORM/Hardware files/Sensors/Temperature/Images/ConductimeterPIC12F675_00.png]

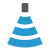

Supplement: Supplementary Data 1 [file mmc1.zip › MONITORING MULTIPLE PARAMETERS IN COMPLEX WATER SCENARIOS USING A LOW COST OPEN SOURCE DATA ACQUISITION PLATFORM/Software files/Data logger/Sketches/Library/ping-arduino/doc/ping.png]

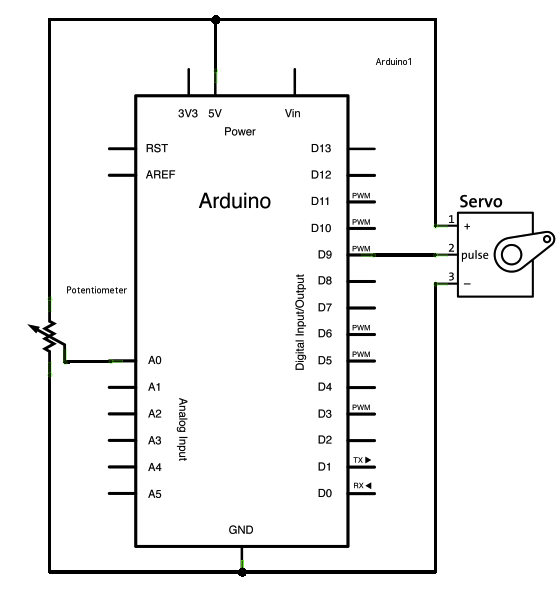

Supplement: Supplementary Data 1 [file mmc1.zip › MONITORING MULTIPLE PARAMETERS IN COMPLEX WATER SCENARIOS USING A LOW COST OPEN SOURCE DATA ACQUISITION PLATFORM/Software files/Data logger/Sketches/Library/Servo/examples/Knob/images/knob_schem.png]

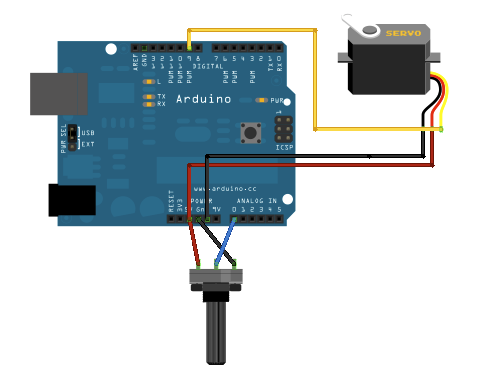

Supplement: Supplementary Data 1 [file mmc1.zip › MONITORING MULTIPLE PARAMETERS IN COMPLEX WATER SCENARIOS USING A LOW COST OPEN SOURCE DATA ACQUISITION PLATFORM/Software files/Data logger/Sketches/Library/Servo/examples/Knob/images/knob_BB.png]

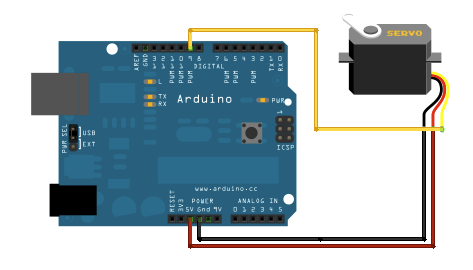

Supplement: Supplementary Data 1 [file mmc1.zip › MONITORING MULTIPLE PARAMETERS IN COMPLEX WATER SCENARIOS USING A LOW COST OPEN SOURCE DATA ACQUISITION PLATFORM/Software files/Data logger/Sketches/Library/Servo/examples/Sweep/images/sweep_bb.png]
