# Supplementary material for: Monitoring multiple parameters in complex water scenarios using a low-cost open-source data acquisition platform
Source: HardwareX. 2023 Nov 28;16:e00492. doi: 10.1016/j.ohx.2023.e00492 (PMC10749909; doi:10.1016/j.ohx.2023.e00492)
Supplement: Supplementary Data 1 [file EMAC-USV-Datalogger.pdf › MONITORING MULTIPLE PARAMETERS IN COMPLEX WATER SCENARIOS USING A LOW COST OPEN SOURCE DATA ACQUISITION PLATFORM/Hardware files/Data logger/EMAC-USV-Datalogger.pdf]

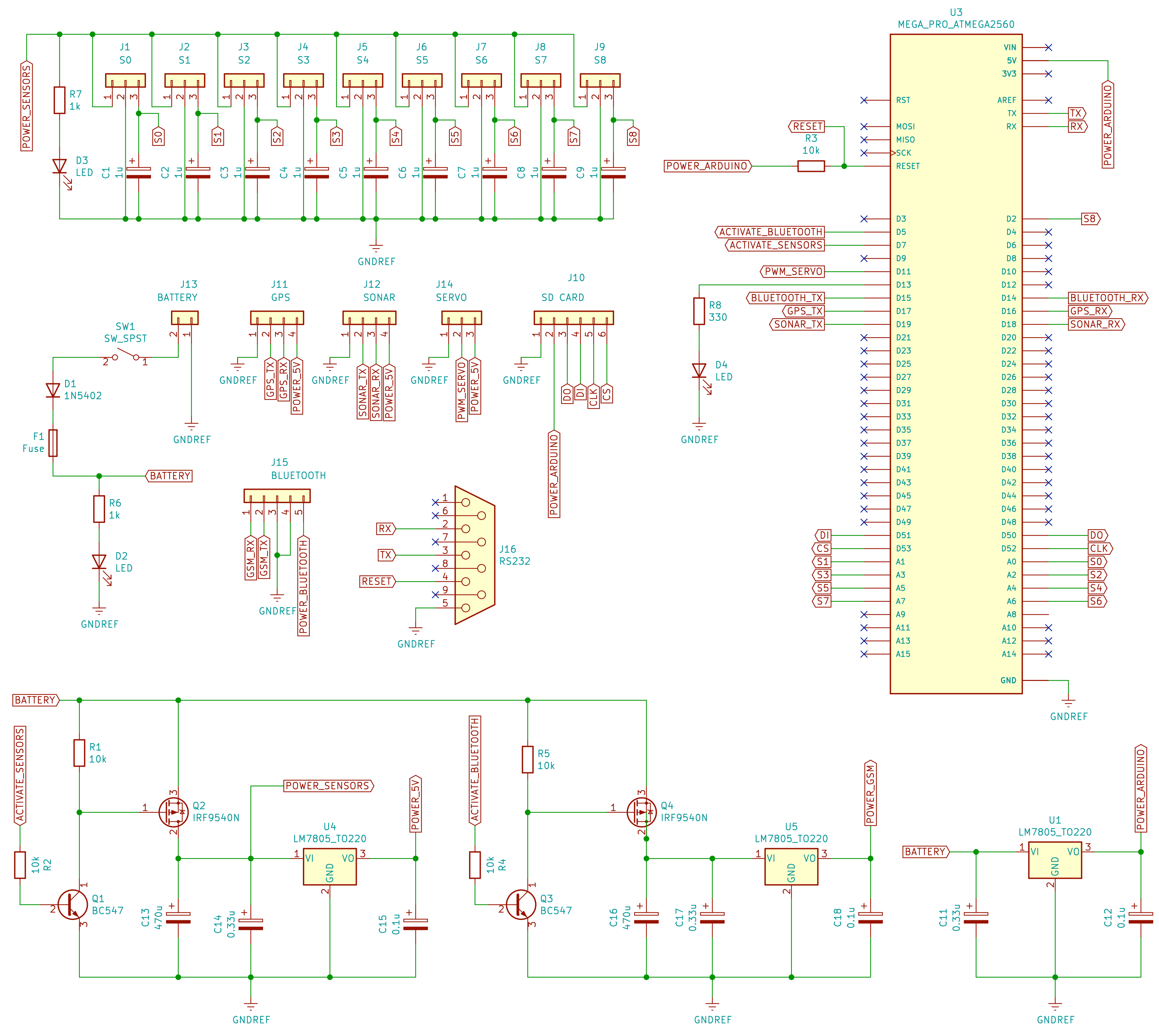

EMAC-IADO

Sheet: /
File: usv.sch

Title: EMAC-USV Datalogger

Size: A3	Date:	Rev: 2
KiCad E.D.A. kicad (5.1.8-0-10_14)		Id: 1/1
