# Supplementary material for: Monitoring multiple parameters in complex water scenarios using a low-cost open-source data acquisition platform
Source: HardwareX. 2023 Nov 28;16:e00492. doi: 10.1016/j.ohx.2023.e00492 (PMC10749909; doi:10.1016/j.ohx.2023.e00492)
Supplement: Supplementary Data 1 [file SSC.pdf › MONITORING MULTIPLE PARAMETERS IN COMPLEX WATER SCENARIOS USING A LOW COST OPEN SOURCE DATA ACQUISITION PLATFORM/Hardware files/Sensors/Suspended solids concentration /SSC.pdf]

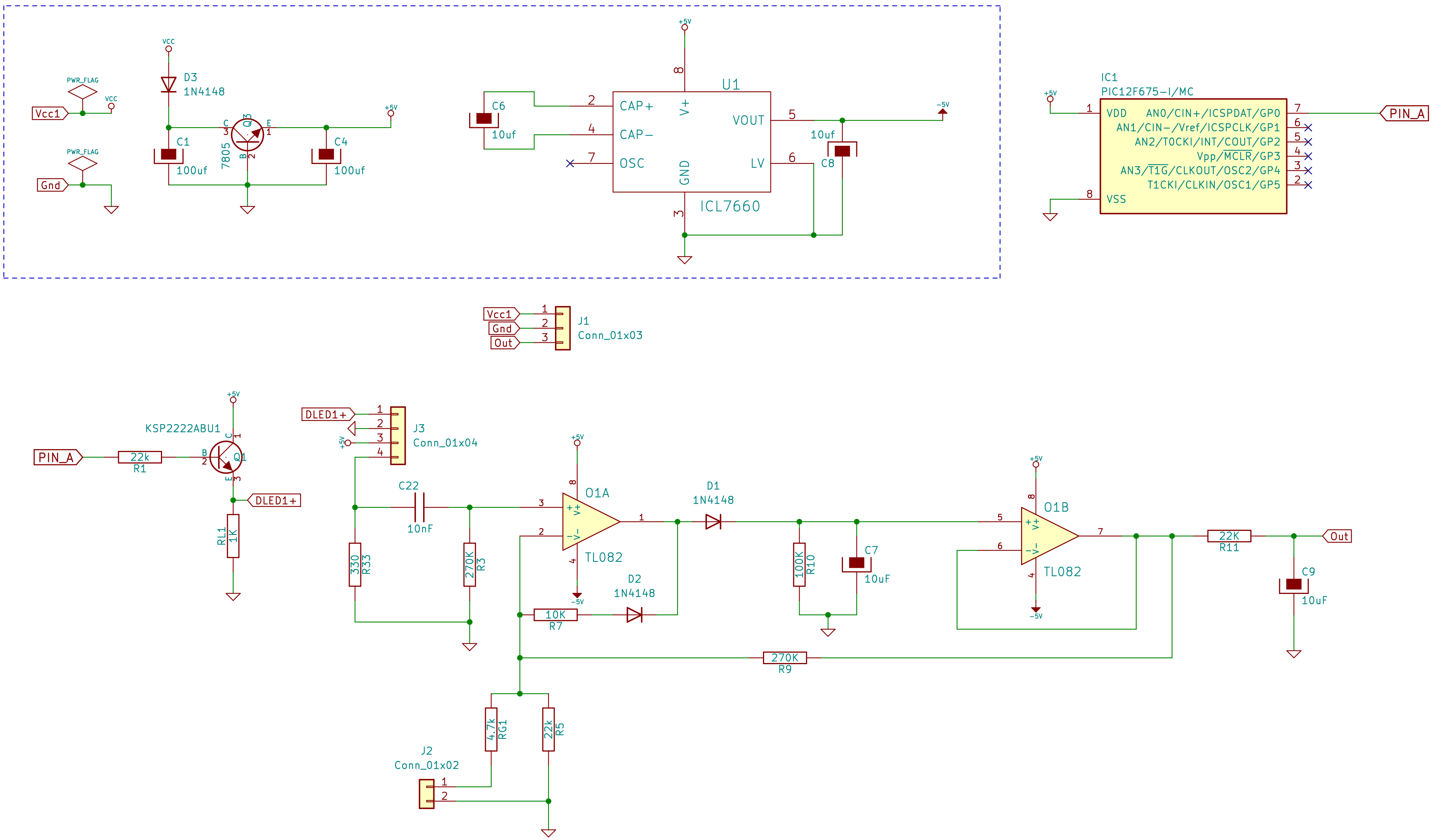

Sheet: /
File: OBS_PIC12F675.kicad_sch

Title:

Size: A4	Date:	Rev:
KiCad E.D.A. kicad (6.0.9-0)		Id: 1/1
